# Phylogeny‐Aware Metabologenomics Accurately Assigns Natural Products to Biosynthetic Gene Clusters

**DOI:** 10.1111/1751-7915.70298

**Published:** 2026-01-14

**Authors:** Judith Boldt, Christoph Porten, F. P. Jake Haeckl, Joachim J. Hug, Fabian Panter, Matthias Steglich, Joachim Wink, Jörg Overmann, Markus Göker, Daniel Krug, Rolf Müller, Ulrich Nübel

**Affiliations:** ^1^ Leibniz Institute DSMZ Microbial Genome Research Braunschweig Germany; ^2^ German Center for Infection Research (DZIF) Partner Site Hannover‐Braunschweig Braunschweig Germany; ^3^ Helmholtz Centre for Infection Research Helmholtz Institute for Pharmaceutical Research Saarland (HIPS) Saarbrücken Germany; ^4^ Department of Pharmaceutical Biotechnology Saarland University Saarbrücken Germany; ^5^ Molecular Systems Biology Unit European Molecular Biology Laboratory (EMBL) Heidelberg Germany; ^6^ Helmholtz Centre for Infection Research Braunschweig Germany; ^7^ Leibniz Institute DSMZ Microbial Ecology and Diversity Research Braunschweig Germany; ^8^ Technical University of Braunschweig Institute of Microbiology Braunschweig Germany; ^9^ Leibniz Institute DSMZ, Bioinformatics Braunschweig Germany

## Abstract

Tens of thousands of biosynthetic gene clusters (BGCs) have been identified in microbial genomes, but the vast majority of associated natural products (NPs) and their underlying biosyntheses remain unknown. Metabologenomics approaches integrate genomic and metabolomic datasets to statistically associate BGCs to their cognate NPs, yet often suggest many false links. Here, we show that incorporating information on the producer strains' phylogeny greatly improves accuracy. We sequenced 72 *Sorangium* spp. genomes (myxobacteria), predicting 2030 BGCs in 265 gene cluster families (GCFs). Mass spectrometry (MS^1^) revealed 99 metabolite families (MFs) from the same strains. Using a phylogeny‐aware statistical analysis, we identified 43 high‐confidence associations between GCFs and MFs, correctly including 89% of previously characterised links and reducing spurious associations by 33‐fold, compared to simple correlational analysis. Our approach identified previously unknown BGCs for rowithocin and an undescribed poly‐glycosylated NP. It also identified a distinct BGC associated with the production of chlorotonil C variants and refined the BGC for maracen. This study demonstrates the effectiveness of phylogeny‐aware metabologenomics as a scalable strategy for NP discovery and biosynthetic pathway elucidation, and provides a roadmap to improved analyses of paired‐omics data towards NP discovery.

## Graphical abstract

We report a statistical approach for associating biosynthetic gene clusters with their cognate metabolites and demonstrate its unprecedented power for scalable natural product discovery. Notably, it drastically reduces spurious associations by explicitly integrating the phylogeny of microbial producer strains. *Source:* https://BioRender.com/rsv6wxn.

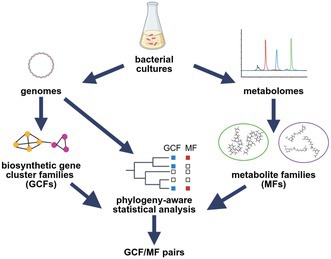

## 1 Introduction

Natural products (NPs) are important sources of biologically active molecules for the discovery of novel drugs (Newman and Cragg 2020). In particular, 55% of antibiotics approved for clinical use since the 1980s are NPs (*n* = 11) or derived from NPs (*n* = 78) (Newman and Cragg 2020). However, the search for novel NPs has been hampered by the abundant re‐discovery of previously known bioactive compounds (Kurita and Linington 2015). The systematic analysis of published NPs has revealed that more recently described compounds often resemble previously discovered structures, while also providing evidence for significant numbers of structurally novel scaffolds still awaiting discovery (Pye et al. 2017). Modern genome sequencing approaches may offer valuable guidance by identifying biosynthetic gene clusters (BGCs) within microbial genomes that encode pathways for the biosynthesis of unique NPs (Zerikly and Challis 2009; Ziemert et al. 2016). Certain types of biosynthetic machineries in microorganisms rely on conserved, modular structures, and the genes involved in a specific biosynthetic pathway are commonly co‐localised on the producer's chromosome (Fischbach and Walsh 2006), facilitating the bioinformatic prediction of BGCs from DNA sequences. Millions of BGCs have thus been annotated in microbial genome sequences (Bagci et al. 2024; Udwary et al. 2024), likely representing tens of thousands of unique biosynthetic pathways (Gavriilidou et al. 2022). However, for the vast majority of these BGCs, the matching products have not been identified. For example, the SMC database (Udwary et al. 2024) currently contains the sequences of 13.2 million BGCs from prokaryotes (https://smc.jgi.doe.gov/; accessed in March 2025). Of these, only 24,440 (0.2%) have significant sequence similarity to any of the 2437 experimentally verified BGCs with known compounds in the manually curated MIBiG database (Zdouc et al. 2025) (mainly from microorganisms; https://mibig.secondarymetabolites.org). Consequently, our abilities to predict the NP biosynthesis encoded by a BGC are presently limited. It is not known how many of the sequenced BGCs encode the production of NPs that constitute new structural families, and which of these may be most useful for future biomedical applications (van der Hooft et al. 2020).

Connecting microbial BGCs to their produced compounds with high confidence requires genetic experiments involving gene knockouts that are correlated to the loss of production (Zerikly and Challis 2009), or the heterologous expression of entire BGCs (Zhang et al. 2019). However, these strategies are time‐consuming and costly, and not feasible for large numbers of BGCs. Assignment of NPs to BGCs can be achieved at increased throughput by integrated statistical analyses of genomic and metabolomic data from strain collectives (Doroghazi et al. 2014; Duncan et al. 2015). This approach, which has been termed ‘metabologenomics’, is based on correlations between the BGC complement and detected metabolites from the same strains (Goering et al. 2016; van der Hooft et al. 2020). It requires the grouping of similar BGCs from different strains into gene cluster families (GCFs) that represent the entities associated with the production of specific metabolite classes (Navarro‐Muñoz et al. 2020). Recent progress in the development of technologies for both DNA sequencing and mass spectrometry, together with significant reductions of costs associated with these analyses, has generated unprecedented opportunities for correlating paired genomic and metabolomic datasets at large scale (Schorn et al. 2021). To move forward, however, a number of challenges are to be met, and among these is a demand for the improvement of statistical methods for charting associations in highly structured datasets (van der Hooft et al. 2020). Importantly, producer organisms are components of a hierarchically structured phylogeny and any statistical test ignoring this non‐independence will overstate the significance of suggested correlations between the organisms' characteristics (Felsenstein 1985). Specifically, the genomes from closely related bacterial strains tend to encode similar sets of BGCs (Chase et al. 2021), and because phylogenetically related organisms share similarity by descent, phylogenetic information needs to be incorporated into the prediction of connections between genetic and phenotypic traits (Barker and Pagel 2005). This requirement has not been addressed in the context of metabologenomics in the past (Doroghazi et al. 2014; Goering et al. 2016; Hjörleifsson Eldjarn et al. 2021; Caesar et al. 2023). As a consequence, these analyses have been compromised by large excesses of incorrect links that required time‐consuming manual verification and thus hindered their application at increased throughput (Leão et al. 2022; Caesar et al. 2023; Louwen et al. 2023).

Here, we have evaluated a phylogeny‐aware statistical analysis to link BGCs and their NPs from 72 myxobacterial strains. Myxobacteria (*Myxococcales*) thrive in soil and a range of other habitats. They are peculiar for their complex lifestyles, involving the cooperative formation of multicellular fruiting bodies and production of desiccation‐resistant myxospores (Reichenbach 1999). Myxobacteria are of great biotechnological interest because they produce diverse NPs with unique structural features and bioactivities (Weissman and Müller 2010; Schäberle et al. 2014; Wang et al. 2024). NPs from myxobacteria with more than 100 distinct basic scaffolds (and several hundred derivatives) have been reported, and novel core structures continue to be discovered (Weissman and Müller 2010; Herrmann et al. 2017; Kostka et al. 2024). The most prolific source of biomedically useful metabolites among myxobacteria is as yet the genus *Sorangium* (Gerth et al. 2003; Hoffmann et al. 2018), which comprises at least nine distinct species (Mohr et al. 2018; Ahearne et al. 2023). To date, the structures of 185 NPs from *Sorangium* strains have been characterised, of which 38 (21%) are produced specifically by this genus, i.e., they have not been found in any other organisms (Hoffmann et al. 2018). Many of these compounds have antibacterial, antifungal, or cytotoxic activities (Gerth et al. 2003; Masschelein et al. 2017). The BGCs encoding the biosynthesis pathways for 17 natural products from *Sorangium* have been published in the MIBiG database, encompassing mostly polyketides and hybrids of nonribosomal peptides (NRPs) and polyketides (Table S1).

**FIGURE 1:**
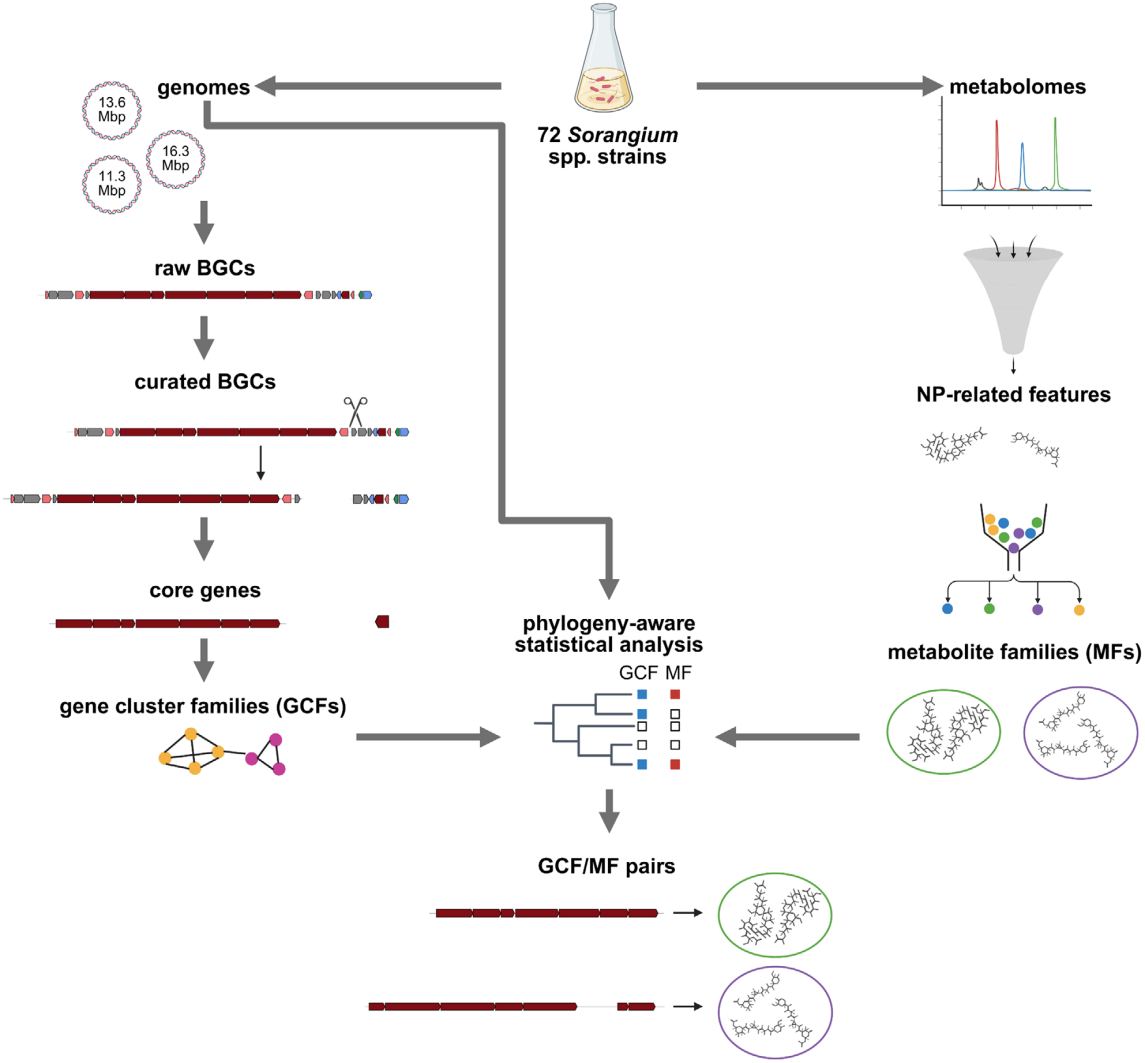
Metabologenomics workflow. All 72 *Sorangium* spp. strains were sequenced using both long‐read (PacBio) and short‐read (Illumina) technologies, resulting in high‐quality genome sequences. These were mined with antiSMASH (Blin et al. 2023) for their biosynthetic gene clusters (BGCs). Potential superclusters were split and BGC regions reduced to their core biosynthetic genes before similar BGCs were grouped into gene cluster families (GCFs) using BiG‐SCAPE (Navarro‐Muñoz et al. 2020). For the metabolomic analysis, UPLC‐MS^1^ measurements of extracts from all 72 Sorangium spp. strains were obtained and processed with MZmine3 (Schmid et al. 2023). The results were filtered using an in‐house database (Hoffmann et al. 2018) for features related to natural products (NP) and grouped by chemical similarity (as reported in the in‐house database) into metabolite families (MFs). For the association of MFs to GCFs, we used a phylogeny‐aware statistical analysis. The genome‐based phylogenetic tree was generated with TYGS (Meier‐Kolthoff and Göker 2019). The figure was created in BioRender (Boldt, J. (2026) https://BioRender.com/2mlew33).

In the present study, we sequenced the complete genomes of 72 *Sorangium* spp. strains, explored them for their BGC complements, and discovered that the vast majority of encoded biosynthetic pathways had not been characterised previously. By combining high‐resolution mass spectrometry (MS^1^) with compound annotations from our in‐house Myxobase database (Hoffmann et al. 2018), the same strains were investigated for their metabolite production under controlled conditions. We then integrated the datasets of genome sequences and experimental metabolomics data to identify statistical associations between BGCs and the production of NPs by applying a maximum‐likelihood approach that explicitly incorporated the genome‐based phylogeny of *Sorangium* (Figure 1). The method is implemented in the software BayesTraits (available at http://www.evolution.reading.ac.uk/BayesTraits.html) and based on the premise that BGCs and NPs which are gained or lost together on multiple occasions along a phylogenetic tree are functionally linked (Barker and Pagel 2005). Considering phylogeny drastically reduced the number of falsely predicted links between GCFs and metabolites. Further, we show that these associations were greatly improved after curation of BGC boundaries on the basis of comparative genomics, and by focusing on core biosynthetic genes for BGC clustering. By applying our phylogeny‐aware metabologenomics approach, we identified previously unreported BGCs for the macrocyclic polyketide rowithocin, a distinct BGC associated with the production of chlorotonil C, a novel family of poly‐glycosylated NPs, and provided additional evidence of BGCs for the polyketide maracen.

## 2 Methods

### 2.1 Bacterial Cultivation and Metabolite Extraction

Cultivation conditions and procedures for metabolite extraction and analysis were described previously (Hoffmann et al. 2018). Briefly, triplicate cultures of *Sorangium* strains were incubated in 100 mL *H* medium (Hoffmann et al. 2018) at 30°C for 14 days in the presence of adsorber resin XAD‐16 (Rohm & Haas), and metabolites were extracted from XAD‐16 by using acetone.

### 2.2 LC–MS Analysis

UPLC‐MS^1^ measurements were performed on a Dionex (Germering, Germany) Ultimate 3000 RSLC system equipped with a Waters (Eschborn, Germany) BEH C_18_ column and a Bruker Daltonics (Bremen, Germany) maXis 4G HR‐qToF mass spectrometer equipped with an Apollo II ESI source, as specified in Data S1. DataAnalysis 5.3 (Bruker Daltonics) was used to recalibrate all MS spectra and to convert the data into the open mzXML format for subsequent processing with MZmine3 (Schmid et al. 2023) (see Table S9). The resulting features were annotated with an in‐house database (Hoffmann et al. 2018) that also provided grouping of metabolite features from similar compounds into metabolite families (MFs) (Table S10). An MF was considered to be present in a strain if at least 3% of the features in the MF and at least one [M + H]^+^ feature were detected in the strain's metabolome.

### 2.3 Genome Sequencing, Assembly, and Annotation

Bacterial genomic DNA was sequenced using both PacBio long‐read and Illumina short‐read technologies. Genome assemblies from long reads were error‐corrected with Illumina data by using BWA 0.6.2 (Li and Durbin 2009) and VarScan 2.3.6 (Koboldt et al. 2012), and annotated with Prokka 1.14.6 (Seemann 2014). Complete genome sequences were submitted to GenBank (https://www.ncbi.nlm.nih.gov/); their accession numbers are indicated in Table S2.

### 2.4 Phylogenetic Analysis

A phylogeny including bootstrap support and species clustering was inferred from whole‐genome sequences by using the Type Strain Genome Server (TYGS) (Meier‐Kolthoff and Göker 2019).

### 2.5 Analysis of Biosynthetic Gene Clusters and Curation

Genome sequences were screened for BGCs using the antiSMASH software version 7.0.0beta1 (Blin et al. 2023), and BGCs were classified into GCFs by using the BiG‐SCAPE framework (version 1.1.5; distance cutoff 0.4) (Navarro‐Muñoz et al. 2020). When BGCs within a GCF were not aligned over their full length, superclusters were split to exclude core biosynthetic genes of neighboring candidate clusters.

### 2.6 Phylogeny‐Aware Correlation Analysis

BayesTraits version 3.0 (available at https://www.evolution.reading.ac.uk/BayesTraits.html) was used with default parameters and no further optimization for detecting phylogenetic correlations between GCFs and MFs, based on the rooted genome‐based phylogeny and a likelihood ratio test (*α* = 0.01; RQ ≥ 0.65, to distinguish correlation from anti‐correlation) between models for independent and dependent evolution (Barker and Pagel 2005). For comparison, Pearson's *χ* ^2^ test for count data (chisq.test, R stats package) was used to detect phylogeny‐unaware correlations between GCFs and MFs in a strain.

### 2.7 Workflow

Detailed descriptions of the steps involved in analysing sequence and metabolome data can be found in Figures S21 and S22, respectively.

## 3 Results

### 3.1 Genomic Diversity

We sequenced, assembled, annotated, and analysed genome sequences from 72 *Sorangium* spp. strains, which had been collected in 36 countries between 1980 and 2000 (Table S2). These strains were selected from a larger strain collection (Hoffmann et al. 2018) to maximise their phenotypic and geographic diversity. The resulting dataset consisted of 62 fully closed genome sequences (including 2 previously published ones (Schneiker et al. 2007; Boldt et al. 2023)), and ten genome sequences consisting of one to three contigs with a completeness of > 93.9% (Table S2).

**FIGURE 2:**
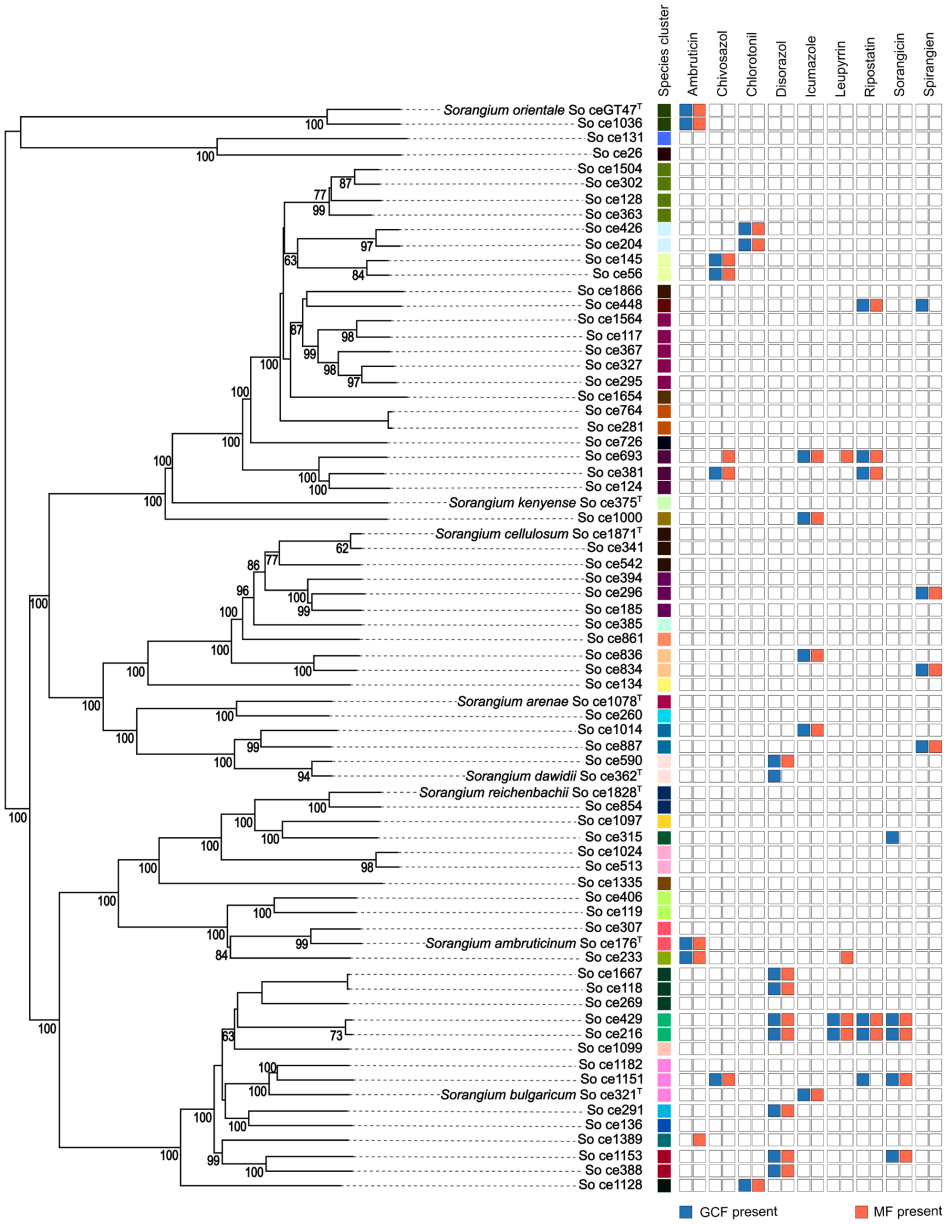
Phylogenetic relationships between the 72 *Sorangium* spp. strains and distribution of known pairs of gene cluster families (GCFs) and metabolite families (MFs). The phylogenetic tree was generated with TYGS (Meier‐Kolthoff and Göker 2019) using the entire genome sequences. Values for 100 pseudo‐bootstrap replicates are indicated at the branches. The 42 proposed species clusters (dDDH < 70%) are indicated in different colours. The presence/absence matrix shows the phylogenetic distribution of GCFs (blue) and MFs (red) from each of the nine known GCF/MF pairs.

Genome‐scale phylogenetic analysis revealed a large diversity among the *Sorangium* spp. strains. There were multiple clades in the phylogenetic tree with digital DNA–DNA hybridization (dDDH) similarities < 70%, suggesting that the set of strains may represent 42 distinct species (Figure 2) (Meier‐Kolthoff and Göker 2019). This number was unexpectedly high, even though the existence of several species within the genus *Sorangium* had previously been proposed based on the diversity of phenotypes and housekeeping gene sequences (Lee et al. 2013; Mohr et al. 2018).

### 3.2 Diversity and Phylogenetic Distribution of BGCs

**FIGURE 3:**
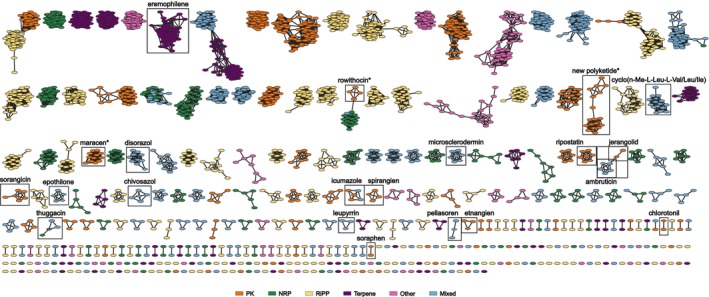
Gene cluster family network. After manual curation of the superclusters, BGCs were grouped into GCFs with BiG‐SCAPE (cutoff, 0.4) as shown here. BGCs from this study are represented as oval nodes, BGCs from MIBiG as diamonds. Node colours represent the different NP classes (see legend). GCFs containing BGCs from MIBiG and the icumazole BGC are highlighted with boxes. The GCFs associated with maracen, rowithocin, and the new poly‐glycosylated family (as proposed by this study) are indicated with boxes and asterisks.

By using the antiSMASH software (Blin et al. 2023) combined with manual curation of ‘superclusters’ (i.e., large BGC regions that contained several BGCs (Medema et al. 2011), see below), we discovered a total of 2432 BGCs in the dataset of 72 *Sorangium* spp. genome sequences, with 21–61 BGCs per genome (Table S3). Classification with BiG‐SCAPE (Navarro‐Muñoz et al. 2020), including the MIBiG database (Terlouw et al. 2023), grouped the predicted BGCs into 489 GCFs (Figure 3). Only 16 (3.3%) of the resulting GCFs included known BGCs from MIBiG (ambruticin and jerangolid BGCs clustered into the same GCF). On the other hand, 187 (38%) GCFs represented singleton BGCs which occurred only once in the entire dataset. Apart from this, the number of BGCs in a GCF varied between two and 60, with a median of four. Both the number and composition of GCFs depended on the parameters used for clustering the BGC sequences. Modifying the cutoff distance from 0.3 (default) to 0.4 or 0.5, for example, changed the total number of GCFs from 548 to 489 and 467, respectively. A cutoff of 0.4 provided the best results regarding the clustering of the 16 GCFs with known BGCs from MIBiG, and therefore was used throughout our analysis.

**FIGURE 4:**
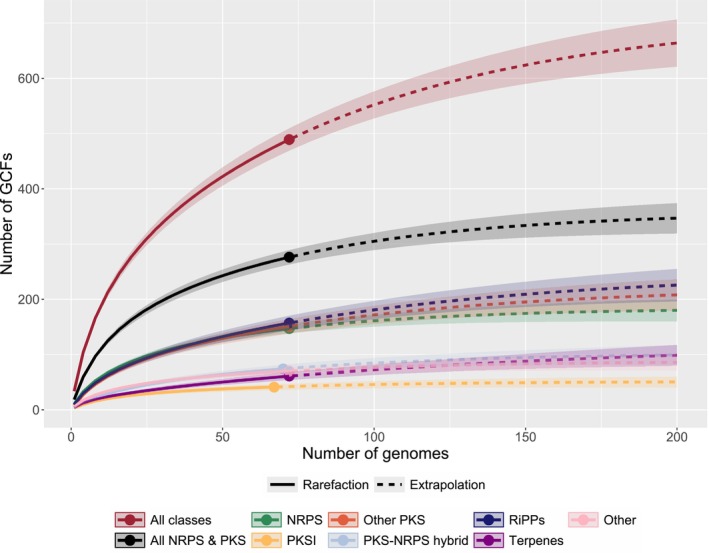
Rarefaction/extrapolation curve. The rarefaction parts of the curves are indicated with continuous lines, the extrapolation parts with dashed lines. The curves represent the analysis with all GCFs (red), the GCFs including NRPSs and PKSs (black), or GCFs corresponding to one of the NP classes. The figure was generated with the R package iNEXT (Hsieh et al. 2016).

The detected BGCs encode diverse pathways for the production of polyketides, NRPs, ribosomally synthesised and post‐translationally modified peptides (RiPPs), terpenes, and other compounds (Table S4). A rarefaction and extrapolation analysis suggested that the investigation of additional genomes from *Sorangium* spp. would lead to the discovery of more GCFs of each of the different classes, but also indicated that with the 72 chosen strains we are covering a large portion of the predicted GCFs (Figure 4). Further increase towards saturation would require a substantial number of additional strains.

Most GCFs were distributed discontinuously across phylogeny, i.e., they occurred in multiple phylogenetic clades of *Sorangium*, and the BGC complements even of closely related strains were not always identical (Figure S1). Accordingly, the coefficient of BGC gain was generally lower than the coefficient of BGC loss (Figure S2), resulting in a very low gain versus loss ratio with a median of 0.016 (median absolute deviation of 0.006) and indicating that throughout *Sorangium* evolution BGCs were only seldom gained and most often lost. For example, the GCF containing the previously reported BGC encoding the synthesis of the ripostatin antibiotics (MIBiG accession BGC0001761) (Fu et al. 2017) contained BGCs from So ce381 and So ce693, but not from the closely related So ce124, even though these three strains are part of the same, as yet unnamed *Sorangium* species (Figure 2). Instead, the GCF comprised BGCs from the more distantly related So ce1151, So ce448, So ce429, and So ce216 which represent *Sorangium bulgaricum* and two other as yet unnamed *Sorangium* species, respectively (Figure 2). Similarly, several other BGCs encoding the biosynthesis of known compounds, such as ambruticin, sorangicin, chivosazol, and disorazol, clustered with BGCs from unrelated strains (Figure 2). The genomes from the closely related strains So ce834 and So ce836 each carried 43 and 45 BGCs, respectively (Table S3), but had only 33 of these in common. This difference between their BGC complements is particularly noteworthy, because these two strains do not only belong to the same species (Figure 2), but they also originated from the very same sample of soil, collected by Reichenbach in October 1990 (unpublished notes by Reichenbach).

### 3.3 Correct Assignment of Known Compounds to Known BGCs

To uncover statistical associations between GCFs and their potential NPs, we developed a metabologenomics approach using a phylogeny‐aware analysis implemented in BayesTraits (Barker and Pagel 2005) for linking the genomic dataset from the 72 *Sorangium* spp. strains to a metabolomic dataset from the same strains. On the genomic side, we used 265 GCFs generated by BiG‐SCAPE (without MIBiG) from 2030 BGCs after curation of the antiSMASH‐predicted BGCs and focusing on core biosynthetic genes (see below). On the metabolomic side, we filtered the large number of metabolite features (> 200,000) detected from the 72 strains down to 99 metabolite families (MFs), with each MF comprising features from similar NPs. As a proof of principle, we first focused our analysis on the nine pairs of NPs with known BGCs that were detected in at least two strains in our dataset. These included eight BGCs from MIBiG (Terlouw et al. 2023) and the recently reported icumazole BGC (Xie et al. 2023) (Table S1). For eight of these nine pairs, our phylogeny‐aware approach associated the NP's MF (Table S10) with the correct GCF, containing all BGCs that were similar to the corresponding published MIBiG BGC (Table 1; Figures S3–S11). In contrast, the NP leupyrrin was not assigned to any GCF because the corresponding BGC occurred exclusively in two closely related isolates (Figure 2), not providing sufficient phylogenetic information. For identifying correlated evolution among pairs of GCFs and MFs, BayesTraits required at least two events of GCF and MF gain or loss in the dataset (e.g., see chlorotonil case, Figure 2). Please note that the two leupyrrin producers had a common ancestor that probably could also produce this NP, and therefore this ability was acquired only once across the entire phylogenetic tree (Figure 2).

**TABLE 1:** Statistical associations of nine known GCF/MF pairs. Results from the phylogeny‐aware analysis (BayesTraits) and a *χ* ^2^ test are compared.

Compound	Phylogeny‐aware (BayesTraits)		Phylogeny ignored (*χ* ^2^)	
	Correct	False positives	Correct	False positives
Ambruticin	Yes	0	Yes	12
Chivosazol	Yes	0	Yes	5
Chlorotonil	Yes	0	Yes	4
Disorazol	Yes	1	Yes	31
Icumazole/noricumazole	Yes	2	Yes	9
Leupyrrin	No	0	Yes	14
Ripostatin	Yes	0	Yes	13
Sorangicin	Yes	0	Yes	25
Spirangien	Yes	0	Yes	0
**Total**	8	3	9	113

### 3.4 Refinement of BGC Boundaries Increased the Proportion of Correct Associations

The correct assignment of the majority of the known NPs to their cognate BGCs was possible only after curation of the raw antiSMASH results. A reported problem in BGC prediction is the cluster boundaries. The antiSMASH tool uses a greedy methodology that can result in the output of ‘superclusters’, which are overly large regions containing several BGCs (Medema et al. 2011). As an illustrative example, in the *Sorangium* sp. So ce834 genome, antiSMASH predicted a large region comprising the BGC for the polyketide spirangien and two further potential BGCs—one encoding a RiPP with a redox‐cofactor, and another one a hybrid of NRP‐metallophore and polyketide (Figure S12). With BiG‐SCAPE, this supercluster was initially grouped into a GCF consisting of 12 BGC regions (Figure S13). Since the clustering did not occur along the spirangien BGCs (blue part of GCF 1.1 (raw BGC regions)), but along the BGCs associated with the production of a RiPP (red part of GCF 1.1), the GCF contained a majority of BGC regions without the spirangien BGC. Accordingly, our statistical analysis did not detect the correct GCF/MF pair for the spirangiens (Table S5). Overall, using the raw antiSMASH BGC regions, only two out of the nine known GCF/MF pairs were associated correctly, with the GCF containing all relevant BGCs (Table S5). For other pairs, the GCFs that were linked to the MFs (e.g., for disorazol and ripostatin) were missing relevant BGCs related to the respective NPs. These missed BGCs were part of superclusters and thus grouped into unrelated GCFs. In two further cases, no correct GCF/MF associations, but rather false positive links were found (leupyrrin and spirangien). To split the superclusters, we developed a novel strategy utilising the presence of similar BGCs throughout different strains in our dataset. If a GCF with raw antiSMASH BGC regions contained larger and smaller regions that only partly overlapped (e.g., see Figure S13), we considered the smaller BGC regions to be individually functional and split the superclusters into several BGCs. Over the 72 *Sorangium* spp. strains, this resulted in 204 additional BGCs. Splitting of superclusters into smaller BGC regions markedly increased the number of correctly associated GCF/MF pairs to six out of nine, with the GCFs comprising all BGCs related to the respective metabolite (Table S5).

### 3.5 Focus on Biosynthetic Core Genes

After curation of BGC superclusters, we focused on the core biosynthetic genes to group BGCs into GCFs, thus deliberately neglecting differences in accessory genes and potentially irrelevant genes within the imprecise boundaries of the BGC regions (antiSMASH classification as ‘other’ genes). This improved the assignment of known GCF/MF pairs to eight out of nine (as reported above). This finding was particularly relevant for the chlorotonils in our study. The strains *Sorangium* sp. So ce204 and So ce426 produced exclusively chlorotonil C1 and C2, whereas *Sorangium* sp. So ce1128 produced chlorotonils A and B3. The published chlorotonil BGC deposited in MIBiG (BGC0001299) corresponds to chlorotonil A and B production and clustered well into one GCF with the BGC region of So ce1128 (Figure S5). However, this GCF did not contain any BGCs from the two strains producing chlorotonil C1 and C2, and thus a statistical association with the chlorotonil MF was not possible. Chlorotonil C1 and C2 have been isolated and characterised previously from *Sorangium* sp. So ce265 (not included in this study) (Jungmann et al. 2015), but no distinct BGC has been published so far. A separate GCF in our analysis comprised two BGCs from the chlorotonil C‐producing *Sorangium* sp. So ce204 and So ce426 that showed a remarkable conservation of core gene synteny, as well as amino acid similarity with the chlorotonil A‐related BGCs from MIBiG and So ce1128, but large differences among the accessory genes (Figure S5). BiG‐SCAPE clustering of these core regions resulted in a single GCF containing the three different chlorotonil‐related BGCs. With this, our phylogeny‐aware metabologenomics approach was able to statistically associate the chlorotonil‐related GCF with the chlorotonil MF, and increase the number of correct GCF/MF associations of known pairs. We therefore confidently report BGCs related to the production of chlorotonil C and propose its biosynthesis (Figures S5 and S14). Similar to the chlorotonil A BGC, the proposed chlorotonil C BGCs contain one halogenase encoding gene *ctoA* and four *trans*AT‐polyketide synthase (PKS) genes (*ctoB*—*ctoE* in MIBiG BGC0001299) followed by genes encoding an ATP‐binding protein and two ABC transporters (Figure S5 and Table S6). The chlorotonil C BGCs lack an orthologue of the methyltransferase encoded by *ctoF*, which has been proposed to catalyse α‐methylation at the C2 position of the circular premature chlorotonil scaffold. In addition, detailed comparison of the published chlorotonil A BGC with the herein newly identified chlorotonil C BGC (from So ce204) reveals three minor deviations in the *trans*AT PKS domain architecture (Figure S15). Firstly, module 1 of the proposed chlorotonil C BGC lacks entirely a DH domain (in contrast to an inactive DH domain in the chlorotonil A BGC), and secondly a duplicate ACP domain at the terminal part of module 3. The third deviation of the chlorotonil C BGC—the lack of a *C*‐methyltransferase (cMT) domain in module 6 encoded by *ctoD*—provides a plausible explanation for the missing α‐methyl group at the position C10. Taken together, the lack of a cMT domain in module 6 and the missing *ctoF* homologue, alongside the different sequence of the *ctoA* homologue in the newly identified chlorotonil C BGC, highlights how even subtle BGC differences can lead to the production of distinct chlorotonil variants. Interestingly, the chlorotonil C BGC region (from So ce204 and So ce426) encompassed an additional nonribosomal peptide synthetase (NRPS) BGC. If the colocalization of both BGC types is coincidental (as observed for many BGCs from *Sorangium*, termed superclusters, see above) and each BGC describes an independent biosynthetic pathway or if both biosyntheses are interconnected is currently under investigation.

### 3.6 Consideration of Phylogenetic Relationships Reduced False Associations

In order to evaluate the impact of phylogeny, we compared our phylogeny‐aware metabologenomics approach (using BayesTraits) to the *χ* ^2^ hypothesis test. The *χ* ^2^‐based analysis only took into account the presence and absence of GCFs and MFs in all strains, ignoring the strains' evolutionary relationships. In contrast, BayesTraits considered the phylogenetic relationships of the producer strains, identifying correlated evolution by statistically comparing a model in which GCF/MF pairs evolved independently to another model in which one character evolved dependent on the state of the other. The analysis with *χ* ^2^ predicted 500 GCF/MF pairs involving 135 GCFs and 77 MFs. The phylogeny‐aware analysis, on the other hand, made a total of 43 predictions over 28 GCFs and 33 MFs (Table S11). Although the *χ* ^2^‐based analysis detected all of the nine known GCF/MF pairs, in contrast to eight with the phylogeny‐aware approach, it also suggested large numbers of false positive links (Table 1). Only 7% (9 of 113) of the *χ* ^2^‐based predictions for the known GCF/MF pairs were correct, whereas the phylogeny‐aware analysis produced 73% (8 of 11) correct associations. In turn, consideration of phylogeny reduced the number of false predictions for known GCF/MF pairs from 99 (*χ* ^2^) to 3 (Table 1). For example, the related chlorotonil producers So ce426 and So ce204 (see Figure 1) had four additional BGCs in common (GCFs 885, 888, 890, and 1591), all of which were incorrectly indicated as being associated with chlorotonil biosynthesis by the *χ* ^2^ analysis (Table 1). The phylogeny‐aware approach made use of the additional information that the distantly related chlorotonil‐producing strain So ce1128 only shared the chlorotonil BGC (GCF 877) with other chlorotonil producers (Figure 1 and Figure S5). Therefore, our metabologenomics analysis was much more useful in linking BGCs and NPs. Random permutation of MFs did not result in correct GCF/MF pairs in either statistical analysis, but rather in one false positive association with the phylogeny‐aware approach and nine false positives with *χ* ^2^ (not shown).

### 3.7 Inference of Novel Pairs of NPs and BGCs

Among the 43 GCF/MF associations predicted by our phylogeny‐aware statistical analysis, there was one involving the maracen MF. Putative genes related to the biosynthesis of maracens have previously been proposed based on the analysis of five *Sorangium* strains (Gemperlein 2014), but no BGC sequence was published. Interestingly, the core biosynthetic genes in the proposed maracen BGC were shown to encode the production of the polyunsaturated fatty acid eicosapentaenoic acid (EPA). The conversion of EPA to maracen was attributed to 18 accessory genes within the proposed BGC, but not further proven (Gemperlein 2014). That previous study (Gemperlein 2014) included strain So ce1128, which was also covered by our dataset. Thanks to our metabologenomics analysis, we were able to link the maracen MF to a GCF containing BGCs from 13 strains, including So ce1128 (Figure S16). These BGCs of type heterocyst glycolipid synthase‐like PKS (*hglE*‐KS, antiSMASH annotation) confirm the reported domain structure of the two core biosynthetic genes (*pfa2* and *pfa3*) (Figure S16, So ce1128 BGC). The majority of these BGCs carried 10 of the 13 accessory genes with known function proposed in the Gemperlein thesis (Table S7) (Gemperlein 2014). The BGC regions predicted in the present study comprised more genes than the ones previously reported, including diverse sets of accessory genes. Hence, our metabologenomics analysis was able to independently confirm the previously proposed maracen BGC and identify additional instances of maracen‐related BGCs over several *Sorangium* strains, thus providing new starting points to further investigate the biosynthesis of maracen (outside the scope of this study).

Another predicted GCF/MF pair involved the NP rowithocin. The chemical structure of rowithocin A has previously been published (Hoffmann et al. 2018), but no information on the underlying BGC is available to date. The rowithocin‐associated GCF from our metabologenomics approach comprised BGCs from *Sorangium* sp. So ce118, So ce1667, So ce887, and So ce136 and So ce375 (Figure S17). The BGCs each contain 11 core biosynthetic genes, which were classified as *trans*AT‐PKS or PKS‐like (encoding a β‐branching module), four additional genes encoding β‐branching modules, one to two genes encoding cytochrome P450 enzymes, and one gene encoding a tyrosine phosphatase family protein strictly conserved and consequently associated with the production of the rowithocin NP family (Figures S17 and S18; Table S8). The candidate consensus rowithocin BGC encodes 18 *trans*AT PKS modules (Figure S18). The standalone AT functionality is encoded by *rowD* as fused tandem AT domains. As with various characterised *trans*AT PKS gene clusters (Piel 2010; Helfrich and Piel 2016), the genes for the *trans*‐acting β‐branching components in the rowithocin biosynthesis are clustered in *rowQ–T*. The overall biosynthetic proposal is in good agreement with the observed chemical structure of rowithocins, despite the irregular nature of the rowithocin biosynthesis contradicting the classical textbook colinearity rules, as observed for several other *trans*AT PKS biosynthetic pathways. Interestingly, the structural similarity of the terminal part of rowithocins with difficidin (Zimmerman et al. 1987) is also reflected in the BGC of rowithocin which shares striking similarity with the difficidin BGC (Chen et al. 2006). Consequently, the three genes *rowN–P* and the encoded modules 13–18 share significant similarity to the genes *difJ–L* (Figure S19). Resembling the difficidin biosynthesis, the phosphorylation of rowithocins remains in some parts elusive; however, it is important to note that different dephosphorylated rowithocin‐related features were detected in the metabolomes of the five strains, but the phosphorylated rowithocin A was not detected in our samples due to its transient nature. Comparison of diagnostic features and in‐source fragmentation patterns from the original producer of rowithocin A (Hoffmann et al. 2018) and the rowithocin‐associated producers in this study confirmed that these rowithocin‐related features are indeed new derivatives (Figure S20), which guided our refinement of the biosynthetic rowithocin proposal (Figure S18).

### 3.8 Metabologenomics Connected Structurally Similar Metabolites

In addition to the correctly predicted known GCF/MF pairs, our phylogeny‐aware metabologenomics approach was able to recognise similarity among NPs, despite them having erroneously been assigned to separate MFs. This was the case for the icumazoles and noricumazoles, which were originally classified as two different MFs, but were both predicted to be associated with the same GCF, which included the BGC from So ce836 known to produce icumazoles (Xie et al. 2023). An evaluation of the biosynthesis of icumazoles (Xie et al. 2023) and the structural similarity between icumazoles and noricumazoles (Barbier et al. 2012) indicated that the same BGC indeed has the potential to produce both compound classes. Consequently, icumazoles and noricumazoles should be considered to belong to the same MF.

**FIGURE 5:**
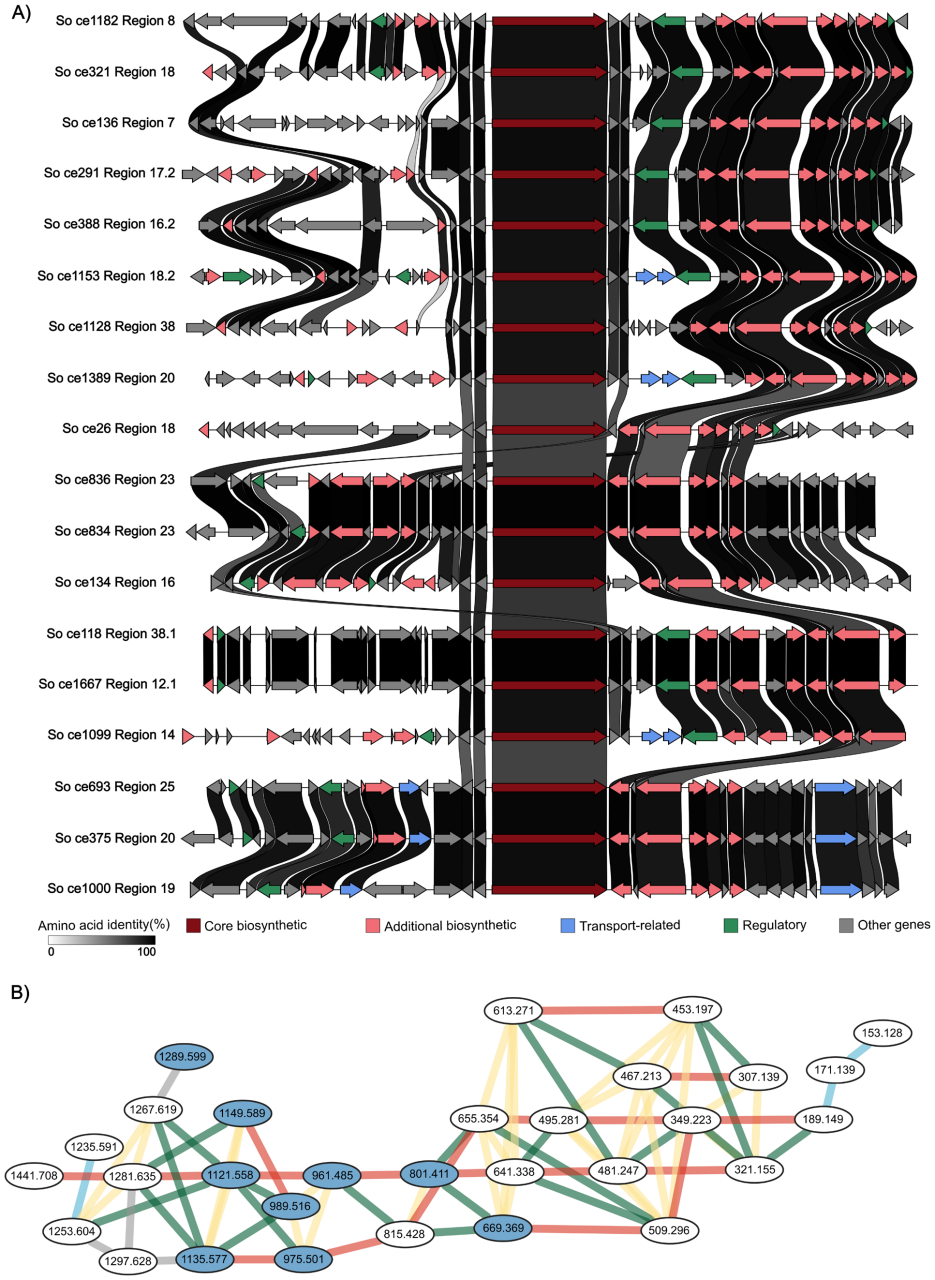
BGCs of a novel poly‐glycosylated MF and molecular network of related MS^1^ in‐source fragments. (A) Representation of the similarity between 18 polyketide‐related BGC regions within the GCF associated with nine uncharacterized MFs. (B) Molecular network of in‐source fragments related to the nine MFs. Identified related ions are described with their m/z values in nodes. Blue coloured nodes correspond to the nine uncharacterized MFs. Edge colours symbolise nature of the losses: (blue) water loss, (green) sugar losses (annotated as either fucose or pentose), (red) defining 160.074 unit loss, (yellow) alkyl losses (CH_2_, C_2_H_4_, C_3_H_6_, C_4_H_8_), (grey) other masses (O, CO_2_, Na adduct, 162.051). The longest chain of six 160.074 units can be followed from 1441.708 to 481.247, interrupted by a methyl loss/addition to 495.281 and two further 160.074 unit losses to 189.149.

Furthermore, our in‐house database included nine MFs that had previously been detected in extracts from both *Sorangium* sp. So ce836 and So ce38. Each of these MFs was detected in 15–17 *Sorangium* strains in our dataset, even though So ce38 was not included in our study. Our metabologenomics analysis consistently associated these compounds with the same GCF (Figure 5A), indicating that they followed a similar phylogenetic distribution across the 72 strains and may therefore be related. This prompted us to investigate these features further. Subsequent analysis of in‐source fragments confirmed their high structural similarity and revealed multiple glycosylations (Figure 5B). Potential related losses in the MS^1^ spectra were mapped manually to a network, which included all previously identified MFs but extended the mass range. Network analysis revealed up to eight putative glycosylations with the same 160.074 unit across the scaffold, which is most likely sugar related (e.g., a methylated deoxy‐hexose), as related losses are annotated as methyl‐ and fucose‐losses. Notably, while some glycosylated compounds are known from *Sorangium*, like the chivosazols (Irschik et al. 1995), epothilones (Wang et al. 2009), sorangiosides (Jansen et al. 1989), and sorangiadenosines (Ahn et al. 2008), multiple glycosylations are unprecedented. Linking the yet uncharacterized MFs to a single GCF using our metabologenomics approach, followed by an analysis of their interrelationships, led to the identification of a highly intriguing MF designated for investigation in future studies.

## 4 Discussion

### 4.1 BGC Diversity and Evolution

In the genomes from 72 *Sorangium* spp. strains, we discovered a total of 489 GCFs, of which the vast majority (> 96%) had not previously been characterised and hence encode unknown pathways. About a third of these GCFs were found only in single genomes in the dataset, and rarefaction analysis suggested that the overall diversity of BGCs in the genus *Sorangium* was even larger. The majority of redundant GCFs were found in multiple phylogenetic clades, indicating limited phylogenetic conservation, and causing limited predictability of the BGC complement at the species level. Even conspecific strains collected from the same soil sample shared only 75% of their BGCs, indicating diversity of biosynthetic capacity to be found over small environmental scales, and corroborating similar findings on *Myxococcus xanthus* (Krug et al. 2008). Similarly, NPs from myxobacteria had previously been reported to display little specificity at the level of species, even though they were largely specific at the genus level (Hoffmann et al. 2018). This non‐clonal distribution of BGCs is likely caused by horizontal transmission between *Sorangium* strains, and probably also between different genera of myxobacteria. While the molecular mechanisms of lateral gene transfer in myxobacteria have received little attention in the past, the co‐localised arrangement of biosynthetic genes on the bacterial genome facilitates their joint transfer into a novel genetic context (Fischbach et al. 2008). Frequent acquisitions of BGCs may be driven by selective advantages conferred by NPs, e.g., by mediating interactions with other organisms, including competitors and predators (Reichenbach 1999). On the other hand, BGC losses may cause little damage, as they do not encode essential housekeeping functions and can be compensated by those BGCs retained.

Sequence variants of BGC regions may correspond to structural variants of the associated metabolites, as e.g., suggested in our analysis of the chlorotonils. Such variants frequently are of biotechnological interest in the search for NPs with improved pharmacological characteristics and for investigations into structure–activity relationships (Naini et al. 2019).

### 4.2 Association of Metabolites and BGCs

The co‐occurrence of BGCs with specific metabolite features in producer strains may provide hints about their functional associations (Doroghazi et al. 2014; Goering et al. 2016). However, closely related *Sorangium* strains share similarity by descent. Since the strains are constituents of a hierarchically structured phylogeny, they cannot be validly considered independent samples from a common distribution, and any statistical test that ignores phylogeny and assumes independence is likely to overestimate the significance of correlations between their genetic and phenotypic traits (Felsenstein 1985). For detecting associations between GCFs and MFs, we therefore applied a statistical method that explicitly incorporated phylogenetic information (Barker and Pagel 2005). As expected, this method found a much smaller number of associations than a simple *χ* ^2^ test. Although the *χ* ^2^‐based analysis correctly predicted all nine known GCF/MF pairs, it also provided a large number of false predictions, and the overall percentage of correct associations was extremely low (7%). In contrast, with the phylogeny‐aware approach we had 73% correct associations and a 33‐fold reduction in the number of false links. Reassuringly, eight of the nine GCFs corresponding to known metabolites (ambruticin, chivosazol, chlorotonil, disorazol, icumazole, ripostatin, sorangicin, and spirangien) were associated with the correct MFs. As an exception, leupyrrin was not assigned to its known, cognate BGC, even though the compound was produced by the two isolates carrying the BGC. Phylogenetic information was insufficient in this case, because these two isolates formed a monophyletic branch in the phylogenetic tree, highlighting that successful associations with the phylogeny‐aware strategy depend on a sufficiently large and diverse collection of strains. In our dataset, the identification of functional links between GCFs and MFs required a minimum of two independent events of gain or loss of these traits in the phylogenetic tree. In theory, such a distribution may be observed in a dataset of at least four strains.

No correct links between known GCF/MF pairs were predicted after random permutation of metabolite features among strains, confirming that the detected associations were robustly based on real signals present in the data.

We predicted 43 significant associations for 28 GCFs and 33 MFs, including links for eight known BGCs. On this basis, we proposed a BGC for the chlorotonil C variants, refined a previously suggested BGC for the production of maracen, and identified for the first time BGCs for rowithocin production and for a previously unidentified NP with highly interesting glycosylation patterns. Further associations comprised GCFs that to date have not been structurally characterised and MFs without knowledge of their biosynthesis. These findings represent a considerable success, given the diverse and error‐prone nature of genomic and metabolomic datasets, the focus on a relatively small sample set of 72 *Sorangium* spp. strains, and their metabolomic analysis under one specific laboratory condition. A definitive proof of biosynthetic links would require the generation of deletion mutants for each BGC, combined with in‐depth metabolomic analyses, which represents a challenging endeavour due to the genetic intractability of many *Sorangium* strains and is therefore beyond the scope of this study (Hug and Müller 2020). However, our results show that analyses of statistical associations may accelerate the identification of valid links between natural products and their cognate biosynthetic genes significantly. Because our phylogeny‐aware approach is suitable for reducing the number of false links and thus the effort required for manual verification, it may be further automated and applied for larger‐scale analyses (Hjörleifsson Eldjarn et al. 2021).

The majority of GCFs remained unassociated to any MFs for various reasons. On the genomic side, the large diversity and limited redundancy of GCFs in the dataset hindered the identification of additional links between GCFs and MFs. Many GCFs occurred in single strains or single clades of closely related strains that hindered the association with our phylogeny‐aware approach. Sequencing additional strains might alleviate this limitation, but saturating the rarefaction curve would require several hundred *Sorangium* genomes (Figure 4). On the metabolomic side, the clustering of metabolite features into MFs was limited to previously explored NPs listed in our in‐house database (Hoffmann et al. 2018) due to the availability of MS^1^ data only, which greatly reduced the space of analysed MFs. For future studies, the use of tandem mass spectrometry (MS^2^) will provide the means to explore entirely unknown MFs by classifying metabolite features according to their fragmentation patterns, without any prior knowledge about their biochemistry (Wang et al. 2016).

Overall, our results confirm that GCFs are indeed useful units for finding correlations with specific MFs, as proposed previously on the basis of data from actinomycetes (Doroghazi et al. 2014; Goering et al. 2016; Navarro‐Muñoz et al. 2020). It should be kept in mind, however, that the number and composition of GCFs depend on the specifics of the clustering algorithm and parameters applied, and the settings may not be equally optimal for different classes of biosynthetic pathways.

Extensive curation was necessary to correct predicted BGC ‘superclusters’ that otherwise hindered statistical associations (Figure S13). In order to split superclusters rationally, a BGC needed to be present in at least two genomes, one of which had to contain a shorter BGC region. Although we relied on manual inspection of BGCs for this analysis, our approach could be automated and probably implemented in software for BGC identification and classification (Nuhamunada et al. 2024), which would enable it to be applied on a larger scale. We recommend using complete genome sequences for this analysis to avoid fragmented or missed BGCs.

Focusing only on the core biosynthetic genes further improved the associations of the known GCF/MF pairs (e.g., chlorotonil‐associated GCF), but in some cases also led to GCFs with erroneously clustered additional BGCs (Figures S3 and S11). Ultimately, improving the classification of BGCs into metabolite‐specific GCFs will benefit from in‐depth analyses of BGC boundaries and relationships between genetic and chemical diversity, guided by a detailed understanding of biosynthetic logic.

## 5 Conclusions

We present an extensive catalogue of BGCs from *Sorangium* spp., which shows that very likely hundreds of NPs remain as yet uncharacterized, even within this well‐studied myxobacterial genus. By using a phylogeny‐aware statistical approach, we identified 43 associations between GCFs and NPs, correctly including 89% of links which had been fully chemically characterised previously. Explicit consideration of strain phylogeny reduced the number of spurious associations for known GCF/MF pairs by 33‐fold, compared to simple correlation analysis. In addition, we devised a rational method and roadmap for curating predicted BGC regions, which was essential for the success of subsequent statistical associations, highlighting the need for further studies into the structure, functionality, and diversity of BGCs. Our results demonstrate that metabologenomics is a powerful tool for accelerating the identification of links between BGCs and NPs, which is a prerequisite for understanding the biosynthetic potential of producer strains, for investigating its genetic regulation, and for using synthetic biology approaches to rationally engineer molecules.

## Author Contributions

**Judith Boldt:** conceptualization (equal), formal analysis (lead), visualisation (lead), writing – original draft (lead), writing – review and editing (equal). **Christoph Porten:** investigation (equal), formal analysis (equal), writing – review and editing (supporting). **F. P. Jake Haeckl:** investigation (equal), formal analysis (equal), writing – review and editing (supporting). **Joachim J. Hug:** formal analysis (equal), writing – original draft (supporting), writing – review and editing (supporting). **Fabian Panter:** investigation (equal), formal analysis (supporting), writing – review and editing (supporting). **Matthias Steglich:** formal analysis (supporting), writing – review and editing (supporting). **Joachim Wink:** investigation (supporting), resources (equal), writing – review and editing (supporting). **Jörg Overmann:** conceptualization (equal), funding acquisition (equal), writing – review and editing (supporting). **Markus Göker:** conceptualization (equal), software (lead), writing – review and editing (supporting). **Daniel Krug:** conceptualization (equal), formal analysis (equal), writing – review and editing (supporting). **Rolf Müller:** conceptualization (equal), resources (lead), funding acquisition (equal), writing – review and editing (supporting). **Ulrich Nübel:** conceptualization (equal), funding acquisition (equal), supervision (lead), writing – original draft (lead), writing – review and editing (lead).

## Funding

This work was supported by Deutsches Zentrum für Infektionsforschung, 09.720.

## Conflicts of Interest

The authors declare no conflicts of interest.

## Supporting information


**Table S1:** Known compounds and known BGCs. Information on the 18 compounds with known BGCs, including 17 from MIBiG and icumazole, detected throughout our genomic data set. This information includes MIBiG accession numbers, biosynthetic class, number of *Sorangium* strains in the GCFs when applying BiG‐SCAPE (including MIBiG) after supercluster splitting, and number of strains in the MF.

**Table S2:** Genome information including sampling information, sequencing information, and GenBank accession numbers.

**Table S3:** BGC information. Number of BGCs per genome according to the different data states—raw antiSMASH results (raw), curated superclusters (curated), core biosynthetic genes (core).

**Table S4:** Classes of biosynthetic gene clusters. Distribution of BGCs and GCFs over the different classes of natural products (NRPS, PKS I, PKS‐NRPS hybrid, other PKS, RiPPs, terpenes, other) as reported by BiG‐SCAPE for the data set with curated superclusters and a cutoff of 0.4.

**Table S5:** Results for the nine known GCF/MF pairs from the BayesTraits analysis with raw antiSMASH results and split superclusters.

**Table S6:** Information on genes, proposed protein functions, and domains for the chlorotonil‐associated BGCs.

**Table S7:** Information on genes, proposed protein functions, and domains for the maracen‐associated BGCs.

**Table S8:** Information on genes, proposed protein functions, and domains for the rowithocin‐associated BGCs.

**Table S9:** MZmine parameter settings.

**Table S10:** Information from the in‐house database used for metabolite annotation.

**Table S11:** Pairs of GCFs and MFs linked through the phylogeny‐aware statistical analysis.

**Data S1:** mbt270298‐sup‐0012‐Supinfo.docx.

## Data Availability

Our R script for performing BayesTraits, the likelihood‐ratio test, and the *χ* ^2^ test can be found in Zenodo (https://doi.org/10.5281/zenodo.17453824). Our input datasets including matrices of GCFs and MFs across 72 Sorangium genomes, a phylogenetic tree, and an MZmine batch file with MZmine parameter settings are available from the same Zenodo repository. Genome sequences were submitted to GenBank (https://www.ncbi.nlm.nih.gov/), see Table S2 for accession numbers. Mass spectrometry (LC–MS) data files were deposited in the MassIVE database (https://massive.ucsd.edu) and assigned accession number MSV000100173.
