# Supplementary material for: Phylogeny‐Aware Metabologenomics Accurately Assigns Natural Products to Biosynthetic Gene Clusters
Source: Microb Biotechnol. 2026 Jan 14;19(1):e70298. doi: 10.1111/1751-7915.70298 (PMC12800573; doi:10.1111/1751-7915.70298)
Supplement: Supplementary file 12 — Data S1: mbt270298‐sup‐0012‐Supinfo.docx. [file MBT2-19-e70298-s011.docx]

**SUPPLEMENTARY INFORMATION**

**Phylogeny-aware metabologenomics accurately assigns natural products to**

**biosynthetic gene clusters**

Judith Boldt^1,2^, Christoph Porten^3,4^, F. P. Jake Haeckl^3^, Joachim J. Hug^5^, Fabian Panter^3,4^, Matthias Steglich^1,2^, Joachim Wink^6^, Jörg Overmann^2,7,8^, Markus Göker^9^, Daniel Krug^3,4^, Rolf Müller^2,3,4^, Ulrich Nübel^1,2,8^

**Methods**

**Bacterial cultivation and metabolite extraction.** Cultivation conditions and procedures for metabolite extraction and analysis were described previously ^1^. Briefly, triplicate cultures of *Sorangium* strains were incubated in 100 mL *H* medium ^1^ at 30°C for 14 days in the presence of 2% (v/v) adsorber resin XAD-16 (Rohm & Haas). Metabolites were extracted from XAD-16 by using 100 mL acetone and shaking for 2 h. The extracts were then filtered, evaporated to dryness under reduced pressure and resuspended in 1.1 mL methanol and stored at -80 °C until measurement. Before analysis, extracts were returned to room temperature, centrifuged for 10 min with 20,000 × *g* and diluted 1:10 in methanol.

**LC-MS analysis.** UPLC-MS^1^ measurements were performed on a Dionex (Germering, Germany) Ultimate 3000 RSLC system equipped with Waters (Eschborn, Germany) BEH C_18_ column (100 × 2.1 mm, 1.7 μm) equipped with a Waters VanGuard BEH C_18_ 1.7 μm guard column. Separation of 1 µL sample was achieved by a linear gradient from (A) H_2_O + 0.1% FA to (B) ACN + 0.1% FA at a flow rate of 600 µL/min and 45 °C. The gradient was initiated by a 0.5 min isocratic step at 5% B, followed by an increase to 95% B in 18 min to end with a 2 min step at 95% B before re-equilibration with initial conditions. UV-vis spectra were recorded by a DAD in the range from 200 to 600 nm. The LC flow was split to 75 μL/min before entering the Bruker Daltonics (Bremen, Germany) maXis 4G HR-qToF mass spectrometer equipped with an Apollo II ESI source. The split was set up with fused silica capillaries of 75 and 100 µm I.D. and a low dead volume tee junction (Upchurch). Mass spectra were acquired in centroid mode ranging from 150-2500 *m*/*z* at a 2 Hz scan rate in centroid mode. Mass spectrometry source parameters were set to 500 V as end plate offset; +4000 V as capillary voltage; nebulizer gas pressure 1 bar; dry gas flow of 5 L/min and a dry temperature of 200 °C. Ion transfer and quadrupole settings were set to funnel RF 350 Vpp.; multipole RF 400 Vpp as transfer settings and ion energy of 5 eV as well as a low mass cut of 300 *m/z*. At the beginning of every LC-MS run, a basic sodium formate solution was injected through a filled 20 µL loop switched into the LC flow. All MS analyses were acquired in the presence of the lock masses (C_12_H_19_F_12_N_3_O_6_P_3_, C_18_H_19_O_6_N_3_P_3_F_2_ and C_24_H_19_F_36_N_3_O_6_P_3_) which generate the [M+H]^+^ ions of 622.0289; 922.0098 and 1221.9906. DataAnalysis 5.3 (Bruker Daltonics) was used to recalibrate all MS spectra using the sodium formate peak and lock masses and to convert the data into the open mzXML format. The mzXML files were processed and aligned using MZmine3 ^2^ in batch mode (see SI Table 9). Only metabolite features that were not present in the medium control and detected in at least four of six replicates were retained. The resulting features were annotated with an in-house database containing information on metabolites previously found in myxobacteria, and filtered for features related to known natural products or potential novel natural products. The in-house database also provided further grouping of metabolite features from similar compounds into metabolite families (MFs). An MF was considered to be present in a strain if at least 3% of the features in the MF and at least one [M+H]^+^ feature were detected in the strain’s metabolome (SI Table 10).

**DNA extraction, genome sequencing, assembly, and annotation.** The genome sequences of *Sorangium* sp. So ce56 and So ce836 have previously been published ^3,4^ and are available at NCBI under the RefSeq accession numbers GCF_000067165 and GCF_028553905, respectively. The other 70 genomes were sequenced using a combination of long-read sequencing (RSII, Sequel II CLR, or Sequel II HiFi technologies from Pacific Biosciences, United States) and short-read sequencing (MiSeq from Illumina, United States). The long-reads were assembled using either the Hierarchical Genome Assembly Process 2 (HGAP2) or HGAP3 ^5^ implemented in SMRT Portal version 2.2.0, or the Microbial Assembly protocol implemented in SMRT Link versions 10 and 11 (for details see SI Table 2). The resulting contigs were trimmed and, if possible, circularized and orientated towards *dnaA* as the start gene. The Illumina-sequenced short reads were preprocessed with fastp version 0.20.1 ^6^ before error-correction of the long-read assembled genomes using Burrows-Wheeler Alignment bwa 0.6.2 (paired-end) ^7^ and VarScan 2.3.6 ^8^. The corrected genome sequences were submitted to NCBI GenBank (accession numbers CP150309 - CP150371, CP150455, NZ_CP012670, NZ_CP012673.1, JBBMOW000000000, JBBMOX000000000, JBFXLY000000000, JBFXLX000000000, JBBMOV000000000, see also SI Table 2). Genome completeness was assessed with CheckM v1.2.2 ^9^. For gene annotation, Prokka 1.14.6 was used ^10^.

**Phylogenetic analysis.** A phylogeny including bootstrap support from 100 replicates was inferred from whole-genome sequences by using the high-throughput version of the Genome BLAST Distance Phylogeny (GBDP) method ^11^ together with FastME ^12^, as implemented in and described for the Type Strain Genome Server (TYGS) ^13^. The species clustering was also obtained from the TYGS with the default digital DNA–DNA hybridization (dDDH) threshold of 70%.

**Analysis of biosynthetic gene clusters and curation.** All sequences of the data set were screened for biosynthetic gene clusters using the antiSMASH software version 7.0.0beta1 ^14^ and relaxed strictness. Please note that RiPP-like BGCs were underestimated in this version of antiSMASH, but this did not further impact our analysis, since RiPP compounds from *Sorangiineae* are currently not included in the in-house database. For classification of BGCs into gene cluster families (GCFs), the BiG-SCAPE framework (version 1.1.5) was used at distance cutoff 0.4 and with the --mix flag ^15^. For initial identification of BGCs with known products from the MIBiG database ^16^, BiG-SCAPE was used with the option '--mibig'. The input data for the statistical analyses however, was generated without the MIBiG database. The splitting of the superclusters was done according to the BiG-SCAPE results with the raw antiSMASH BGC regions. If a GCF contained BGCs that were not aligned over the whole BGC regions, but only along a candidate cluster, we split the respective regions into the candidate clusters, specifically excluding core biosynthetic genes of neighboring candidate clusters. Please note that the further curation of the BGCs by reducing them to the core biosynthetic genes, led to the loss of several terpene- and RiPP-related BGCs due to genes with less than 1,000 bp length. Since BGC prediction with antiSMASH requires genes to be at least 1,000 bp long, these short BGCs could not be reanalyzed and were thus excluded from the BiG-SCAPE clustering, resulting in an overall number of BGCs lower than with the raw antiSMASH-predicted BGC regions or the regions after supercluster splitting.

**Phylogeny-unaware correlation analysis.** Pearson’s χ^2^ test for count data (chisq.test), as implemented in the R stats package, was used to detect phylogeny-unaware correlations between pairs of discrete (binary) traits, i.e., the presence or absence of a particular GCF in a strain and the production of a metabolite feature belonging to a MF, or the lack thereof. A correlation between a GCF and an MF was considered significant, if the p-value of the χ^2^ test was ≤0.01.

**Phylogeny-aware correlation analysis.** BayesTraits version 3.0 was used for detecting phylogenetic correlations between GCFs and MFs ^17^. These analyses were performed using the BayesDiscrete ML method with the rooted genome-based phylogeny as the reference tree and default parameters. The likelihood ratio between models for independent and dependent evolution, respectively, was tested against a χ^2^ distribution with one degree of freedom and α=0.01 ^17^. Only when this likelihood ratio test indicated a significantly better performance of the dependent model was a correlated evolution assumed.

**Detailed biosynthesis proposal for rowithocin.** A new member of a macrocyclic phosphorylated polyketide termed rowithocin was discovered in a systematic metabolite survey of ~ 2,300 myxobacterial strains, where derivatives of rowithocin were observed exclusively in strains belonging to the genus *Sorangium* ^1^. The BGC for rowithocin was not yet reported. However, a structurally related natural product family termed (oxy)difficidin had been identified in *Bacillus subtilis* earlier ^18^, and the BGC was confirmed in 2006 ^19^.

Our metabologenomics analysis associated the rowithocin MF with a GCF containing BGCs from the myxobacterial strains So ce118, So ce1667, So ce887, and So ce136 and So ce375 (SI Figure 17). Detailed comparison of these BGCs reveals that 10 core *trans*AT PKS biosynthesis genes (in red), five genes encoding β-branching modules (purple), one to two genes encoding cytochrome P450 enzymes (green) and one gene encoding a tyrosine phosphatase family protein (green) are strictly conserved and associated with the production of the rowithocin natural product family (*rowA–T*, SI Figure 18). The identified rowithocin-BGC present in So ce375 displays an additional gene homolog encoding a tyrosine phosphatase protein (*rowK_1_*), and a different genetic architecture of *rowJ* and *rowL* (SI Figure 17). While the enlarged *rowJ* homolog in So ce375 still features significant homology to all the other *rowJ* genes, the severely truncated *rowL* homolog of So ce375 is not aligning with the other *rowL* homologs. The candidate consensus rowithocin BGC encodes 18 *trans*AT PKS modules (SI Figure 18). The standalone AT functionality is encoded by *rowD* as fused tandem AT domains, and the genes for the *trans*-acting β-branching components in the rowithocin biosynthesis are clustered locally (*rowQ–T*).

The proposed assembly line (SI Figure 18) starts with the loading of butyryl CoA, whereas all other extender units are exclusively incorporating malonyl CoA building blocks. As it has been described for other *trans*AT PKS biosynthetic pathways, *C*-methylations at the α-position of the tethered polyketide backbone are catalyzed by distinct *C*-methyltransferases (cMT) domains. Consequently, the four identified α-methyl branches at C4, C14, C20, C26 of rowithocin A, are likely formed by distinct cMTs. The presence of four cMTs in total at module 2, 5 ,11 and 17 is in good agreement with the observed chemical structure of rowithocin A and guided us to provide a plausible biosynthesis proposal for the non-textbook biosynthesis of the rowithocins. Since the rowithocin biosynthesis features two non–elongating modules, namely module 7 (encoded by *rowI*) and module 15 (encoded by *rowN*), the number of modules incorporating extender units between C26 and C20 (cMTs on module 2 and 5), C14 and C4 (cMTs on module 11 and 17) fits well with the observed carbon length of the polyketide backbone, whereas the presence of six modules between C20 and C14 (cMTs on module 5 and 11) suggests at least one module skipping event; therefore we hypothesize that module 8 is skipped. Interestingly module 8 and module 10 are highly similar, not only in their domain organization but also in the amino acid sequence of the domains. The absence of KR domains – which would be required to catalyze the incorporation of the corresponding reduced β-keto function in modules 3 and 10 – highlights the irregular nature of rowithocin biosynthesis. This deviates from the classical textbook collinearity rules, as has been observed in several other *trans*AT PKS biosynthetic pathways.

Interestingly the structural similarity between the terminal part of rowithocin and difficidin is also reflected in the similarity of their respective BGCs (SI Figure 19). The three genes *rowN–P* and the encoded modules 13–18 share significant similarity to the genes *difJ–L* (SI Figure 19). In addition, the generation of olefinic moieties between C6 to C13 strongly resembles the biosynthetic logic of the difficidins by employing dehydrating type A and type B bimodules. Furthermore, the phosphorylation and proposed post PKS addition of a hydroxyl function at C16 of the rowithocins resembles the proposed decoration of the difficidins.

**Supplementary Tables.**

**SI Table 1**. Known compounds and known BGCs. Information on the 18 compounds with known BGCs, including 17 from MIBiG and icumazole, detected throughout our genomic data set. This information includes MIBiG accession numbers, biosynthetic class, number of Sorangium strains in the GCFs when applying BiG-SCAPE (including MIBiG) after supercluster splitting, and number of strains in the MF.

**SI Table 2**. Genome information including sampling information, sequencing information, and GenBank accession numbers.

**SI Table 3**. BGC information. Number of BGCs per genome according to the different data states - raw antiSMASH results (raw), curated superclusters (curated), core biosynthetic genes (core).

**SI Table 4**. Classes of biosynthetic gene clusters. Distribution of BGCs and GCFs over the different classes of natural products (NRPS, PKS I, PKS-NRPS hybrid, other PKS, RiPPs, terpenes, other) as reported by BiG-SCAPE for the data set with curated superclusters and a cutoff of 0.4.

**SI Table 5**. Results for the nine known GCF/MF pairs from the BayesTraits analysis with raw antiSMASH results and split superclusters.

**SI Table 6.** Information on genes, proposed protein functions, and domains for the chlorotonil-associated BGCs.

**SI Table 7.** Information on genes, proposed protein functions, and domains for the maracen-associated BGCs.

**SI Table 8.** Information on genes, proposed protein functions, and domains for the rowithocin-associated BGCs.

**SI Table 9.** MZmine parameter settings.

**SI Table 10.** Information from the in-house database used for metabolite annotation.

**SI Table 11.** Pairs of GCFs and MFs linked through the phylogeny-aware statistical analysis.

**References.**

1 Hoffmann, T. *et al.* Correlating chemical diversity with taxonomic distance for discovery of natural products in myxobacteria. *Nat Commun* **9**, 803, doi:10.1038/s41467-018-03184-1 (2018).

2 Schmid, R. *et al.* Integrative analysis of multimodal mass spectrometry data in MZmine 3. *Nat Biotechnol* **41**, 447-449, doi:10.1038/s41587-023-01690-2 (2023).

3 Boldt, J. *et al.* Bursts in biosynthetic gene cluster transcription are accompanied by surges of natural compound production in the myxobacterium *Sorangium* sp. *Microb Biotechnol* **16**, 1054-1068, doi:10.1111/1751-7915.14246 (2023).

4 Schneiker, S. *et al.* Complete genome sequence of the myxobacterium *Sorangium cellulosum*. *Nat Biotechnol* **25**, 1281-1289, doi:10.1038/nbt1354 (2007).

5 Chin, C. S. *et al.* Nonhybrid, finished microbial genome assemblies from long-read SMRT sequencing data. *Nat Methods* **10**, 563-569, doi:10.1038/nmeth.2474 (2013).

6 Chen, S., Zhou, Y., Chen, Y. & Gu, J. fastp: an ultra-fast all-in-one FASTQ preprocessor. *Bioinformatics* **34**, i884-i890, doi:10.1093/bioinformatics/bty560 (2018).

7 Li, H. & Durbin, R. Fast and accurate short read alignment with Burrows-Wheeler transform. *Bioinformatics* **25**, 1754-1760, doi:10.1093/bioinformatics/btp324 (2009).

8 Koboldt, D. C. *et al.* VarScan 2: somatic mutation and copy number alteration discovery in cancer by exome sequencing. *Genome research* **22**, 568-576 (2012).

9 Parks, D. H., Imelfort, M., Skennerton, C. T., Hugenholtz, P. & Tyson, G. W. CheckM: assessing the quality of microbial genomes recovered from isolates, single cells, and metagenomes. *Genome Res* **25**, 1043-1055, doi:10.1101/gr.186072.114 (2015).

10 Seemann, T. Prokka: rapid prokaryotic genome annotation. *Bioinformatics* **30**, 2068-2069, doi:10.1093/bioinformatics/btu153 (2014).

11 Meier-Kolthoff, J. P., Auch, A. F., Klenk, H. P. & Göker, M. Highly parallelized inference of large genome‐based phylogenies. *Concurrency Computation* **26**, 1715-1729 (2014).

12 Lefort, V., Desper, R. & Gascuel, O. FastME 2.0: A comprehensive, accurate, and fast distance-based phylogeny inference program. *Mol Biol Evol* **32**, 2798-2800, doi:10.1093/molbev/msv150 (2015).

13 Meier-Kolthoff, J. P. & Göker, M. TYGS is an automated high-throughput platform for state-of-the-art genome-based taxonomy. *Nat Commun* **10**, 2182, doi:10.1038/s41467-019-10210-3 (2019).

14 Blin, K. *et al.* antiSMASH 7.0: new and improved predictions for detection, regulation, chemical structures and visualisation. *Nucleic Acids Res* **51**, W46-W50, doi:10.1093/nar/gkad344 (2023).

15 Navarro-Muñoz, J. C. *et al.* A computational framework to explore large-scale biosynthetic diversity. *Nat Chem Biol* **16**, 60-68, doi:10.1038/s41589-019-0400-9 (2020).

16 Terlouw, B. R. *et al.* MIBiG 3.0: a community-driven effort to annotate experimentally validated biosynthetic gene clusters. *Nucleic Acids Res* **51**, D603-D610, doi:10.1093/nar/gkac1049 (2023).

17 Barker, D. & Pagel, M. Predicting functional gene links from phylogenetic-statistical analyses of whole genomes. *PLoS Comput Biol* **1**, e3, doi:10.1371/journal.pcbi.0010003 (2005).

18 Zimmerman, S. B. *et al.* Difficidin and oxydifficidin: novel broad spectrum antibacterial antibiotics produced by *Bacillus subtilis*. I. Production, taxonomy and antibacterial activity. *J Antibiot (Tokyo)* **40**, 1677-1681, doi:10.7164/antibiotics.40.1677 (1987).

19 Chen, X. H. *et al.* Structural and functional characterization of three polyketide synthase gene clusters in *Bacillus amyloliquefaciens* FZB 42. *J Bacteriol* **188**, 4024-4036, doi:10.1128/JB.00052-06 (2006).

20 Xie, F. *et al.* Insights into the biosynthesis of icumazole, unveiling a distinctive family of crotonyl-CoA carboxylase/reductase. *Cell Reports Physical Science* **4**, 101394 (2023).

21 Jungmann, K. *et al.* Two of a kind--the biosynthetic pathways of chlorotonil and anthracimycin. *ACS Chem Biol* **10**, 2480-2490, doi:10.1021/acschembio.5b00523 (2015).

22 Gemperlein, K. Biosynthesis and heterologous production of polyunsaturated fatty acids from myxobacteria. Doctoral thesis, Universität des Saarlandes, Saarbrücken, Germany, doi:10.22028/D291-23027 (2014).


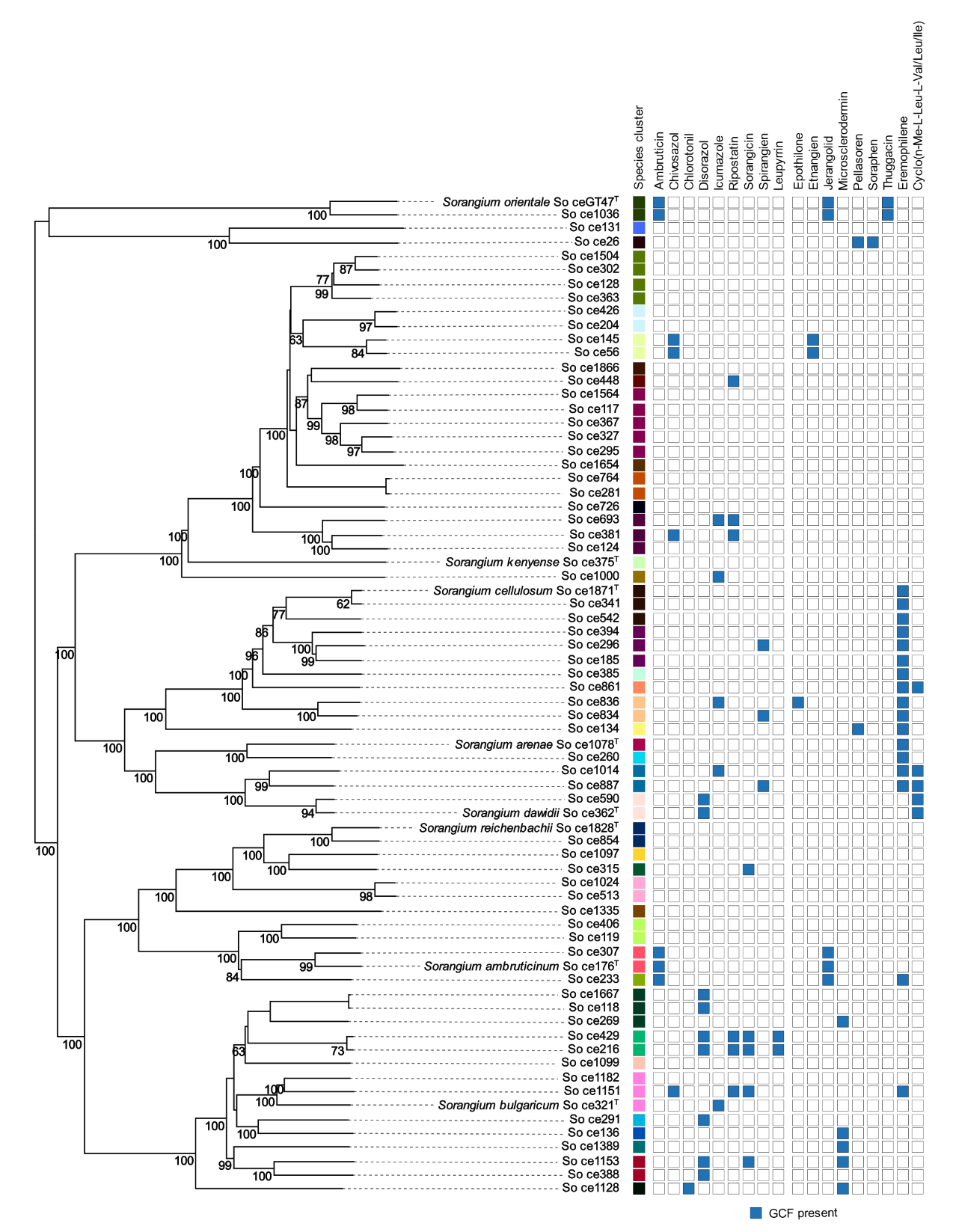


**SI Figure 1.** **Phylogenetic distribution of known gene cluster families (GCFs) across the 72 Sorangium spp. strains.** The phylogenetic tree is as shown in Figure 2. The presence/absence matrix shows the presence of the 17 GCFs (from the BiG-SCAPE analysis (c0.4) after supercluster splitting) with known BGCs from MIBiG, plus the recently published icumazole BGC.


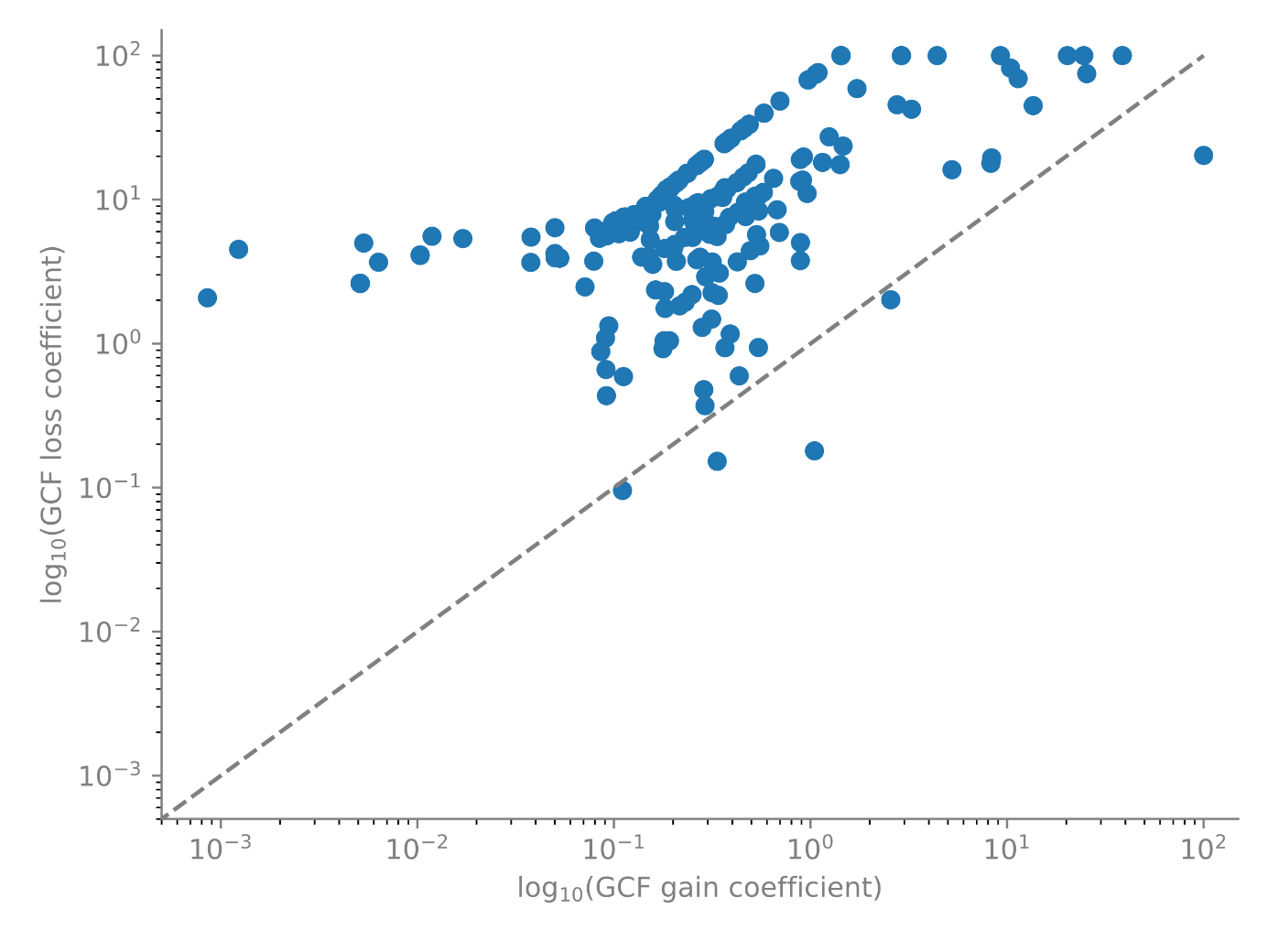


**SI Figure 2.** **Rate coefficients of GCF gain and loss.** For each GCF, the rate coefficients of it to be gained or lost over the 72 Sorangium strains were estimated with BayesTraits and plotted against each other. For most GCFs, the loss coefficient is higher than the gain coefficient. This indicates an overall low presence of the GCF and a high phylogenetic distance of the strains with GCF presence. On the other hand, small loss and gain coefficients would correspond to a GCF presence in few, but closely related strains. A small loss and high gain coefficient would result in a widespread GCF presence over many of the strains, whereas high loss and high gain would represent a GCF present in few strains that are very distantly related.


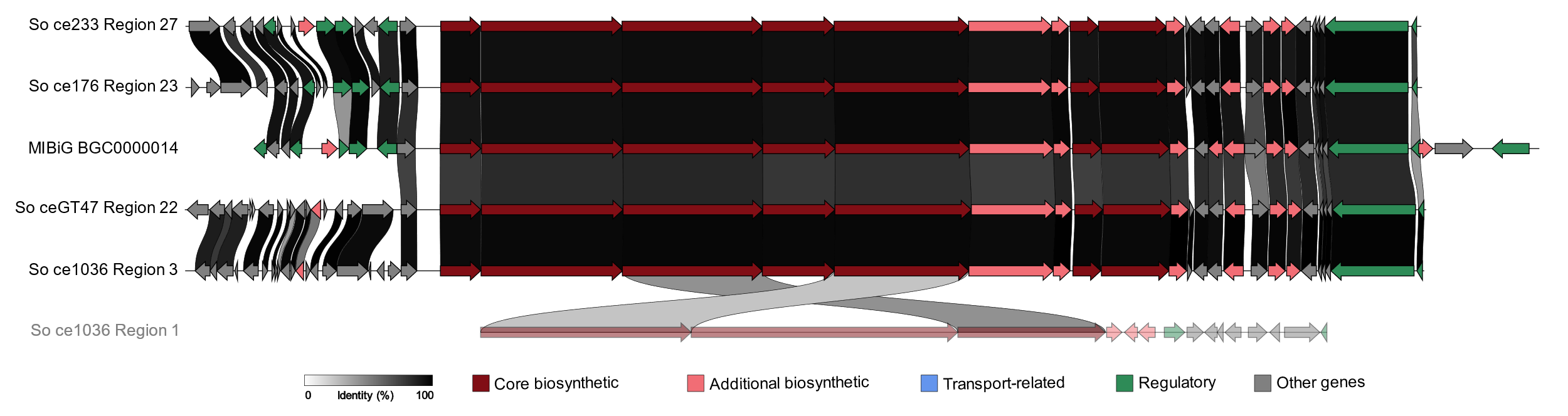


**SI Figure 3.** **Ambruticin-associated BGCs.** Representation of the similarity between the five BGC regions within the ambruticin-associated GCF and the published MIBiG BGC for ambruticin (BGC0000014). Please note that BGC region 1 from Sorangium sp. So ce1036 (in lighter colors) was included in the ambruticin-associated GCF by BiG-SCAPE, but shows low similarity to the published ambruticin BGC and will not produce an ambruticin compound.


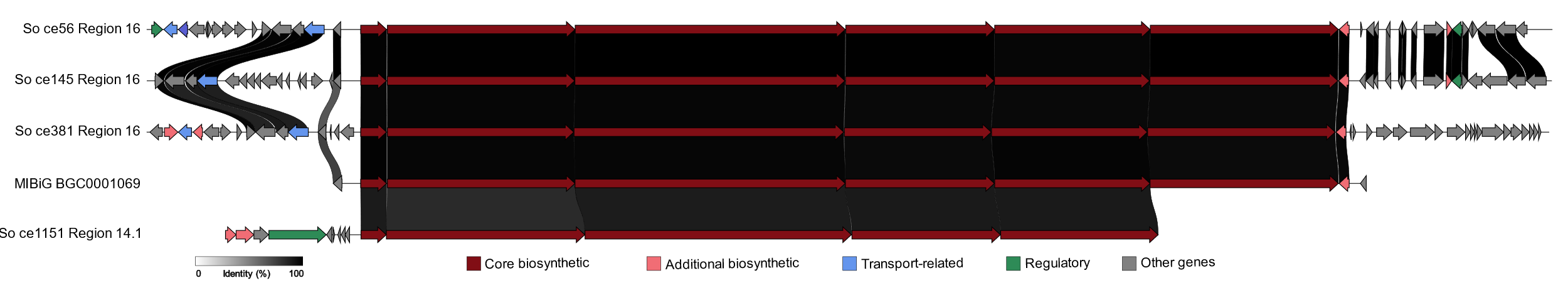


**SI Figure 4.** **Chivosazol-associated BGCs.** Representation of the similarity between the four BGC regions within the chivosazol-associated GCF and the published MIBiG BGC for chivosazol (BGC0001069). BGC region 14.1 from Sorangium sp. So ce1151 originally belonged to a supercluster. The suffix 1 indicates that the represented part is the left most section of the supercluster after the splitting.


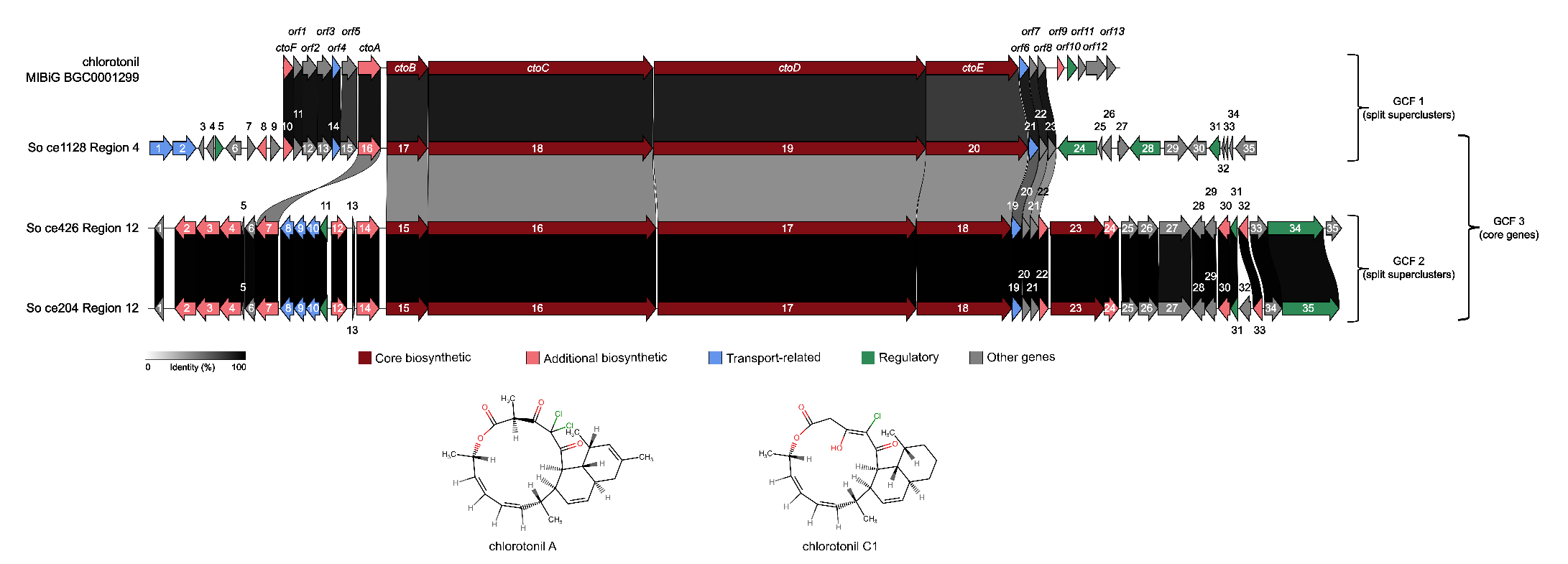


**SI Figure 5.** **Chlorotonil-associated BGCs.** BiG-SCAPE clustered the published MIBiG BGC for chlorotonil A (BGC0001299) and BGC region 4 from Sorangium sp. So ce1128 into one GCF (GCF 1) after supercluster splitting. The BGC regions 12 from Sorangium sp. So ce204 and So ce426 (potentially related to chlorotonil C) were clustered into a separate GCF (GCF 2). The core biosynthetic genes between all four BGC regions show high amino acid identity (indicated by grey scale (see legend)). Considering only the core biosynthetic genes and not including MIBiG BGCs, BiG-SCAPE clustered region 4 from So ce1128 and regions 12 from So ce204 and So ce426 into one GCF (GCF 3). Further information on gene length, biosynthetic type, proposed function, and proposed domains is provided in (SI Table 6). The chemical structures for chlorotonil A and chlorotonil C1 are represented below the BGC regions.


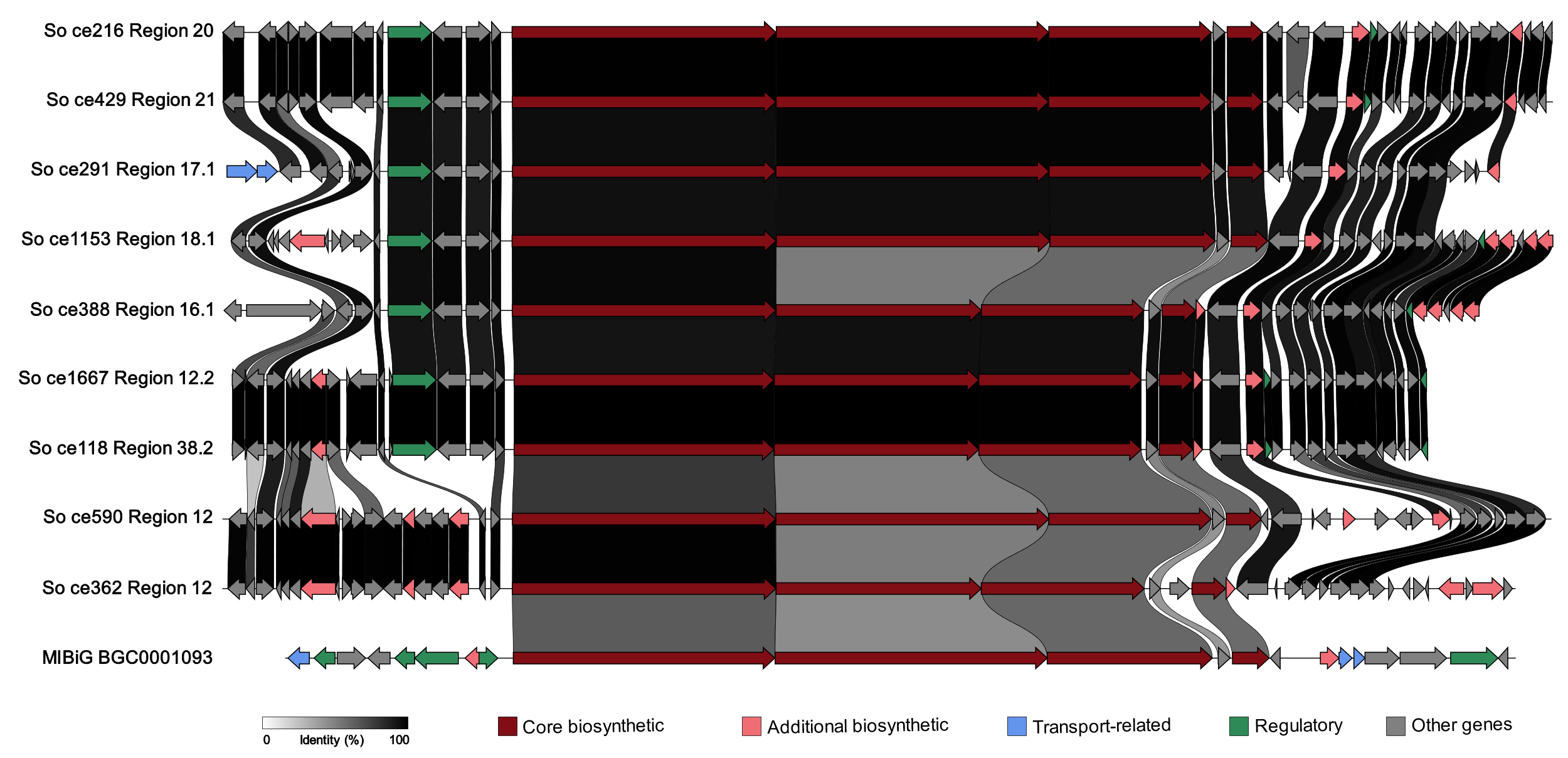


**SI Figure 6.** **Disorazol-associated BGCs.** Representation of the similarity between the nine BGC regions within the disorazol-associated GCF and the published MIBiG BGC for disorazol A (BGC0001093). All BGC regions that were originally parts of superclusters are indicated with a suffix number after the BGC number. The suffix numbering proceeds from left to right (e.g. suffix 1 represents the left most part of a supercluster).


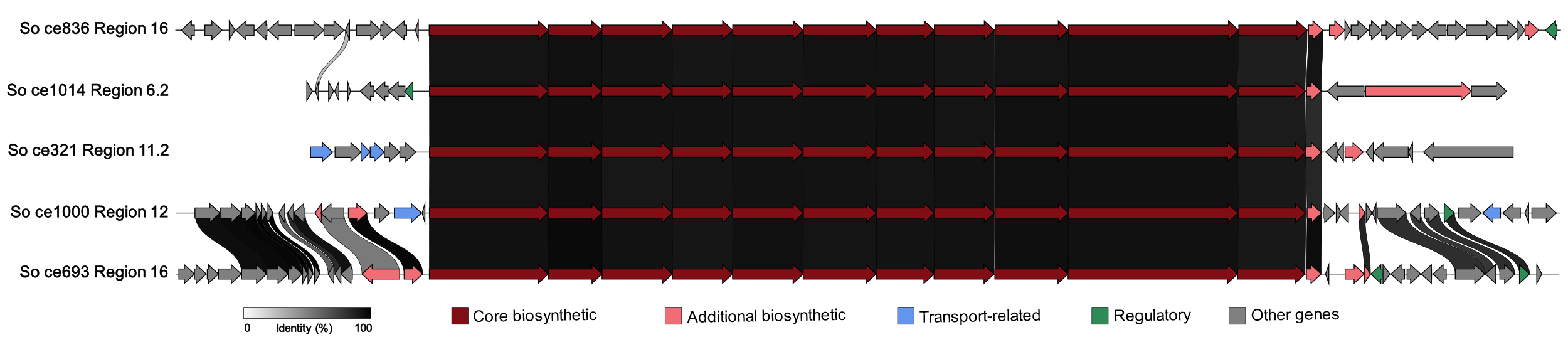


**SI Figure 7.** **Icumazole-associated BGCs.** Representation of the similarity between the five BGC regions within the icumazole-associated GCF. The BGC region 16 from Sorangium sp. So ce836 corresponds to the recently published icumazole BGC ^3,20^. All BGC regions that were originally parts of superclusters are indicated with a suffix number after the BGC number. The suffix numbering proceeds from left to right (e.g. suffix 1 represents the left most part of a supercluster).


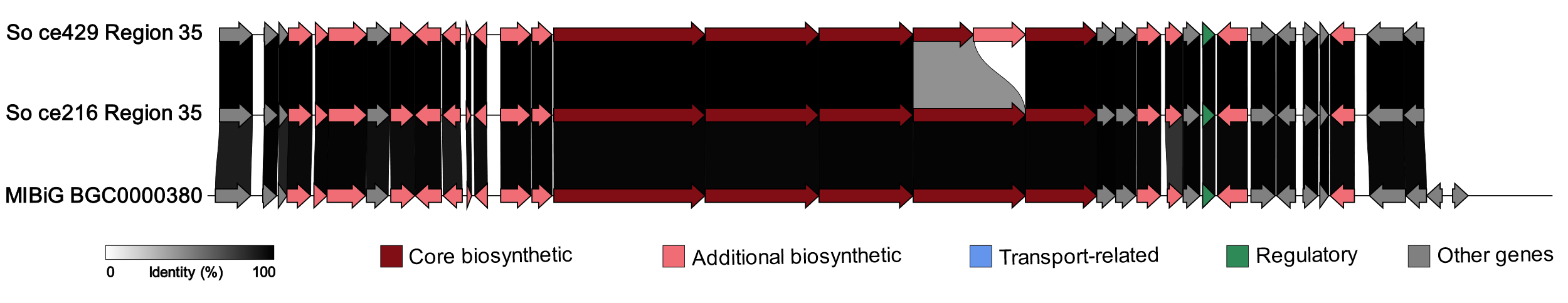


**SI Figure 8.** **Leupyrrin-associated BGCs.** Representation of the similarity between the two BGC regions within the leupyrrin-associated GCF and the published MIBiG BGC for leupyrrins A1, A2, B1, B2, C, and D (BGC0000380).


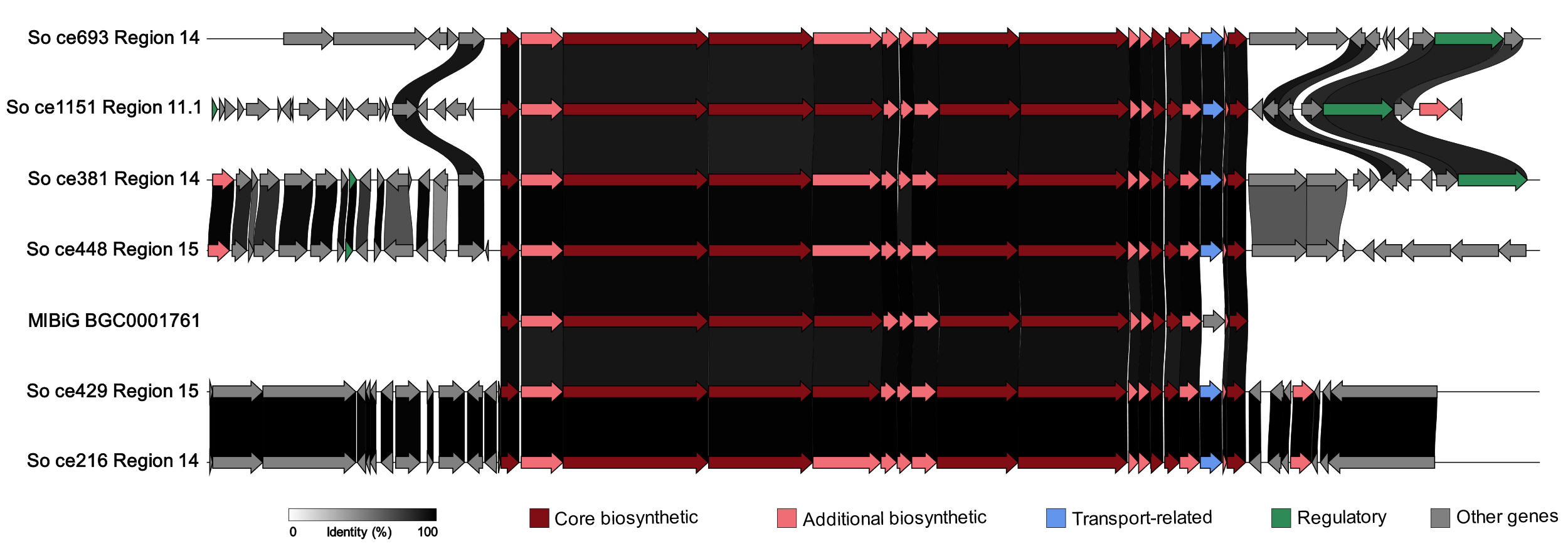


**SI Figure 9.** **Ripostatin-associated BGCs.** Representation of the similarity between the six BGC regions within the ripostatin-associated GCF and the published MIBiG BGC for ripostatins A, B, and C (BGC0001761). BGC region 11.1 from Sorangium sp. So ce1151 originally belonged to a supercluster. The suffix 1 indicates that the represented part is the left most section of the supercluster after the splitting.


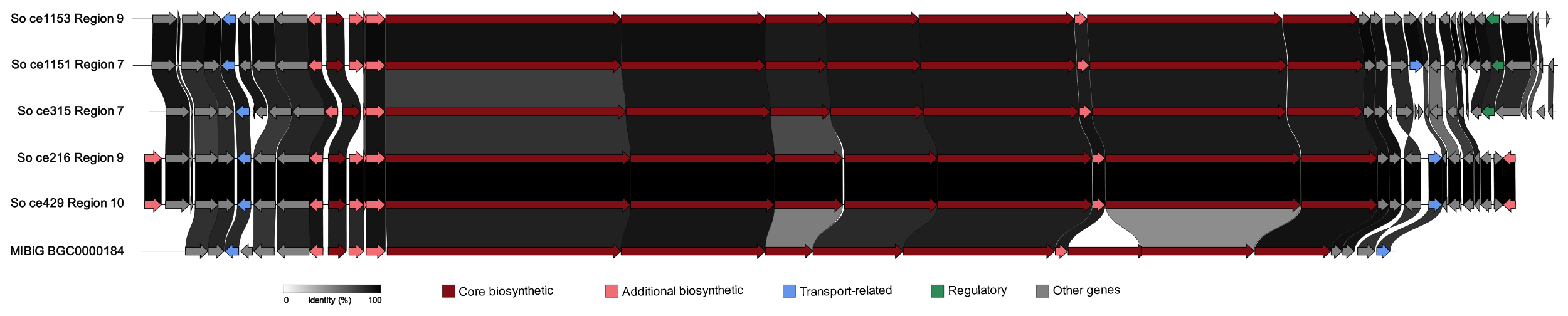


**SI Figure 10.** **Sorangicin-associated BGCs.** Representation of the similarity between the five BGC regions within the sorangicin-associated GCF and the published MIBiG BGC for sorangicin A (BGC0000184).


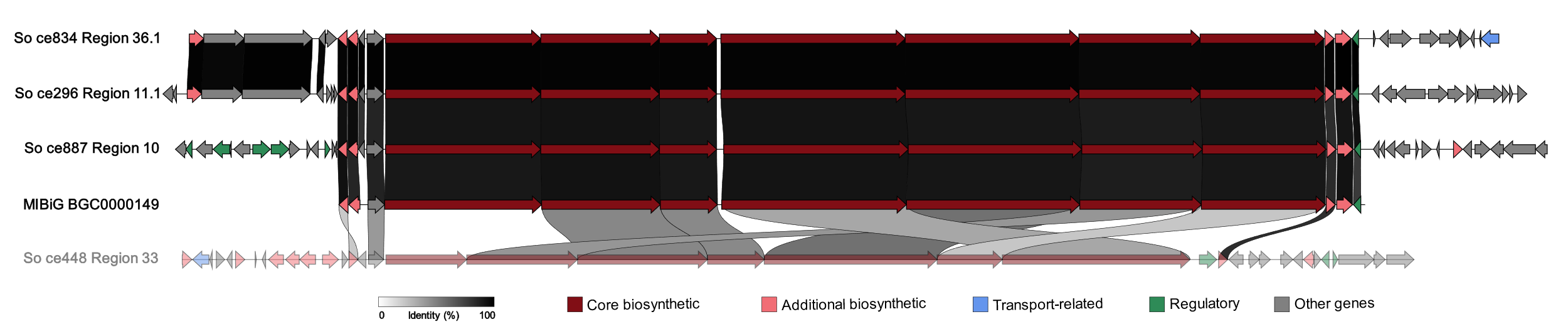


**SI Figure 11.** **Spirangien-associated BGCs.** Representation of the similarity between the four BGC regions within the spirangien-associated GCF and the published MIBiG BGC for spirangien O (BGC0000149). Genes are colored according to the antiSMASH coloring scheme (see legend). Please note that BGC region 33 from Sorangium sp. So ce448 (in lighter colors) was included in the spirangien-associated GCF by BiG-SCAPE, but shows low similarity to the published spirangien BGC and will most likely not produce a spirangien compound. Accordingly, no spirangien compound was detected in the metabolome of So ce448.


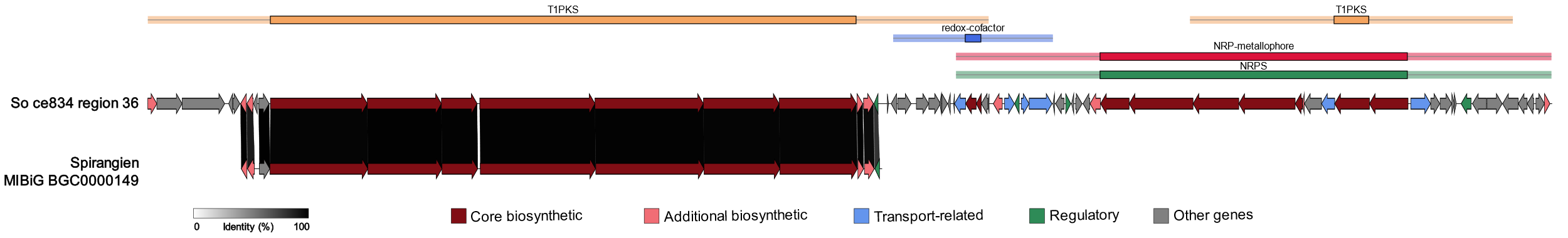


**SI Figure 12.** **Supercluster containing a spirangien-related BGC predicted by antiSMASH in So ce834.** The upper BGC region represents the predicted region 36 from So ce834, the lower BGC region is the spirangien O BGC from MIBiG (BGC0000149). In the BGC region from So ce834, the left T1PKS subcluster corresponds to the blue parts in SI Figure 13 and represents the spirangien-related BGC. The middle redox-cofactor subcluster corresponds to the red parts in SI Figure 13, and the NRP-metallophore subcluster on the right, containing NRPSs and a T1PKS, corresponds to the green parts in SI Figure 13.


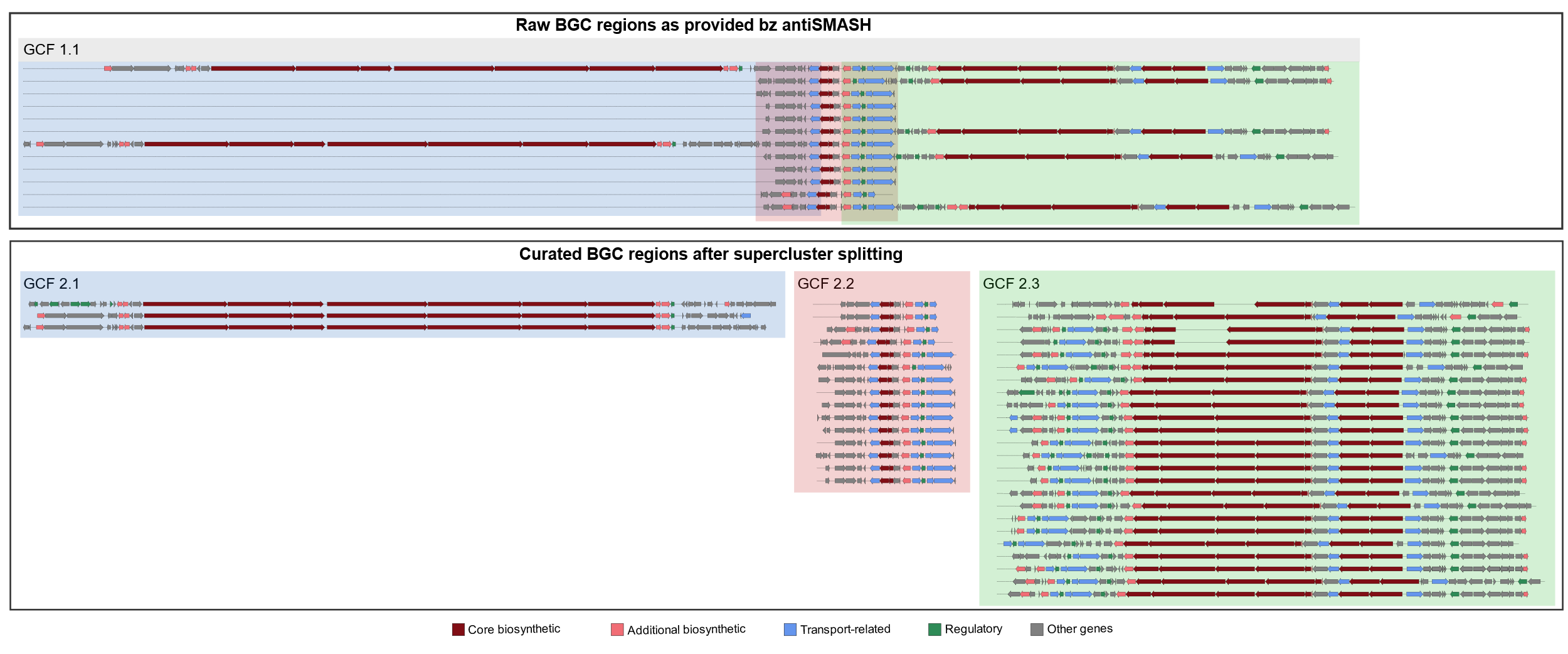


**SI Figure 13.** **Curation of a supercluster.** (Upper panel) Raw BGC regions as provided by antiSMASH were clustered into a single GCF by BiG-SCAPE. The clustering occurred along the red-shaded part of the BGC regions. The GCF contains superclusters spanning also the blue and the green part of the regions. (Lower panel) Splitting of the superclusters along the boundaries of the blue, red, and green subregions resulted in three separate GCFs (2.1, 2.2, 2.3) that include more members than the original GCF 1.1. BGCs in these three GCFs are linked to the production of spirangien (blue), a RiPP (red), and an NRP-metallophore/PK-hybrid (green), respectively.

**SI Figure 14. Identified chlorotonil C BGC and proposed biosynthesis of chlorotonil C1 and C2.** The chlorotonil C biosynthesis features strong resemblance to the biosynthetic pathway of the chlorotonil variants A and B ^21^.

**SI Figure 15. Comparison of the chlorotonil A and C *trans*AT module architecture.** Module architecture of the *trans*AT core biosynthetic genes *ctoC*, *ctoD*, and *ctoE* of the chlorotonil A-related BGC from the original producer strain *Sorangium* sp. So ce1525 ^21^, and of the newly proposed chlorotonil C-related BGC from *Sorangium* sp. So ce204.


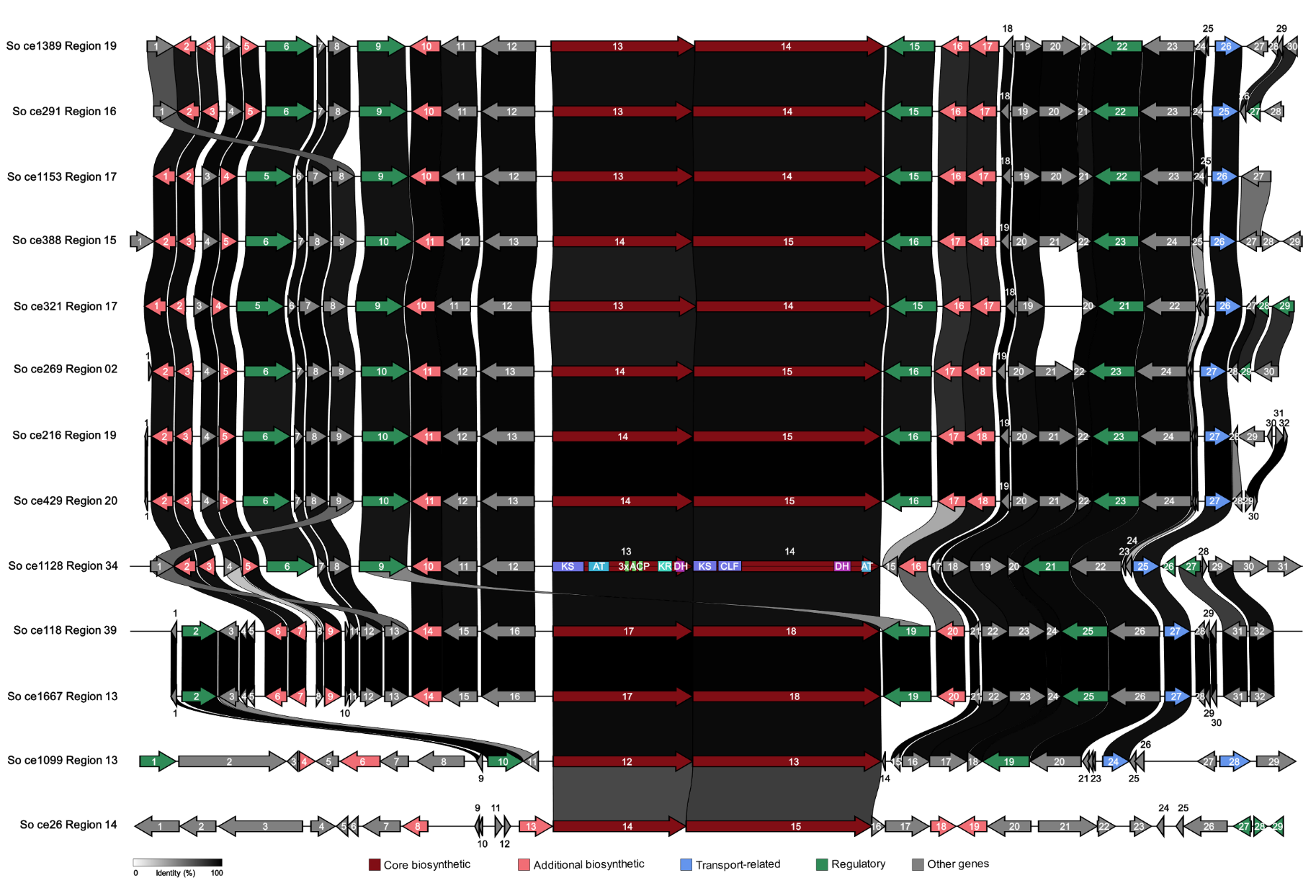


**SI Figure 16.** **Maracen-associated BGCs.** Representation of the similarity between the 13 BGC regions within the maracen-associated GCF. The domains corresponding to the reported domain structure of the maracen-associated core biosynthetic genes (pfa2 and pfa3) ^22^ are indicated in genes 13 and 14 of the BGC region 34 in Sorangium sp. So ce1128. Further descriptions on gene length, biosynthetic type, proposed function, and proposed domains is provided in SI Table 7.


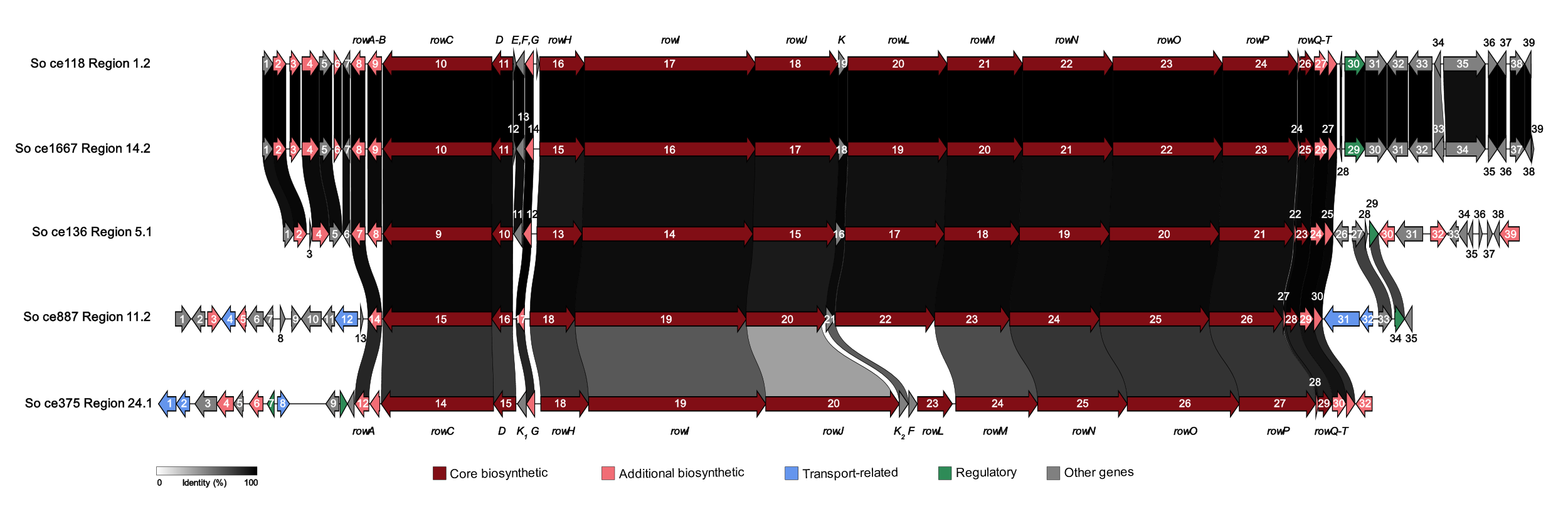


**SI Figure 17.** **Rowithocin-associated BGCs.** Representation of the similarity between the five BGC regions within the rowithocin-associated GCF. All BGC regions were parts of superclusters, as indicated by the suffix of the BGC number. The suffix numbering proceeds from left to right (e.g. suffix 1 represents the left most part of a supercluster). Further descriptions on gene length, biosynthetic type, proposed function, and proposed domains is provided in (SI Table 8).


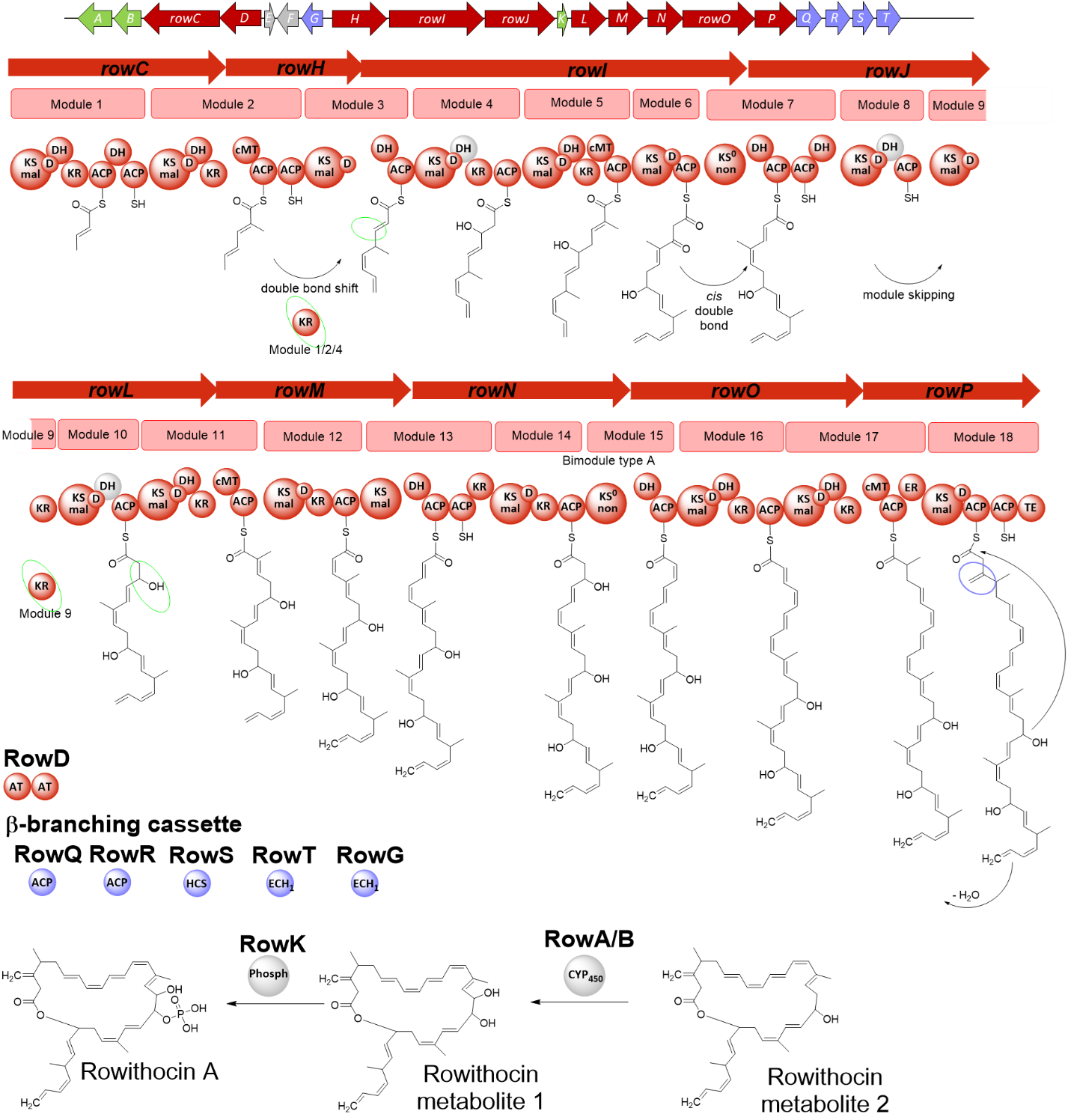


**SI Figure 18. Identified BGC and proposed biosynthesis of rowithocin A and deduced chemical structure of novel rowithocin derivatives.**

**SI Figure 19. Terminal module comparison of the proposed rowithocin BGC and the difficidin BGC.**

**
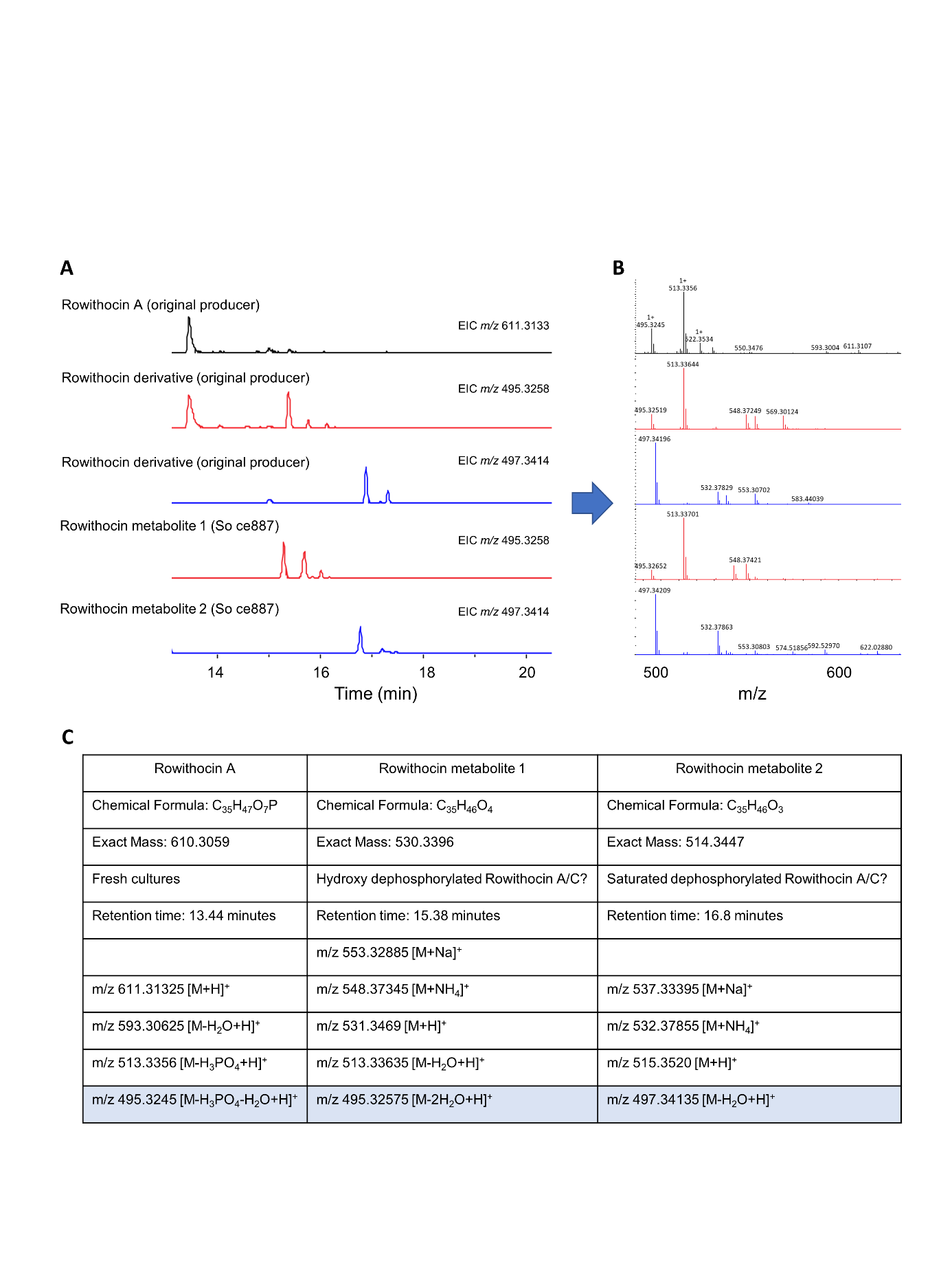
**

**SI Figure 20. Comparison of rowithocin-related features and in-source fragmentation.** (A) Comparison of diagnostic features from the original rowithocin A producer ^1^ and *Sorangium* sp. So ce887 (this study) shows good overlap of the associated features that lead to the discovery of rowithocin A (extracted ion chromatograms, EICs). (B) MS spectra of each of the EICs presented in panel (A) show that m/z 495.3258 and m/z 497.3414 are in-source fragments related to the [M+H]^+^ and other adducts associated with the rowithocin BGC in our dataset. (C) Chemical formulas of rowithocin A (purified, structure elucidated previously) and rowithocins metabolite 1 and metabolite 2 (proposed) and their associated diagnostic ions. Blue background indicates the m/z values used for EIC comparisons. For the elucidated and predicted structures see SI Figure 18.


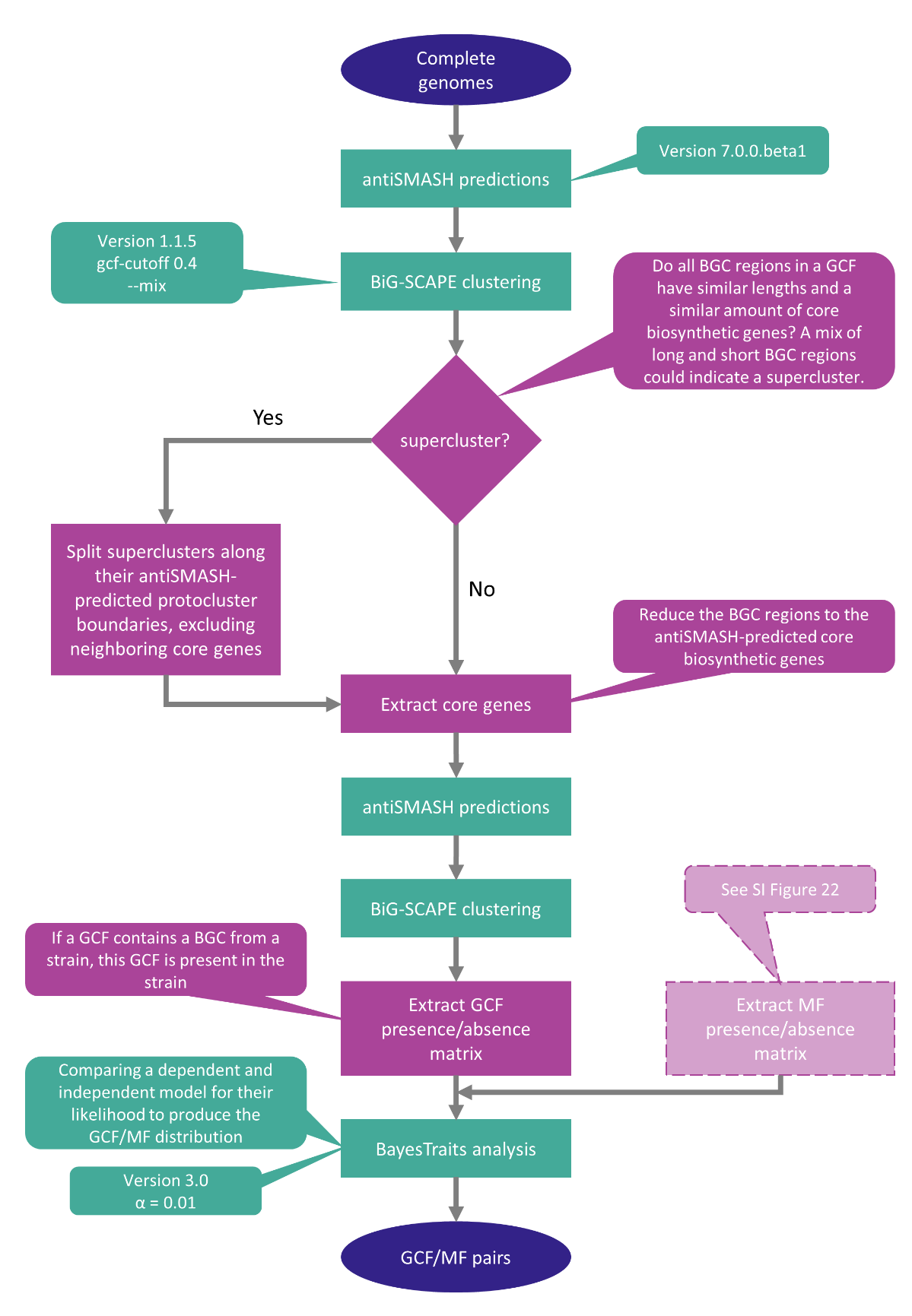


**SI Figure 21.** Genomic workflow of the metabologenomics approach. The individual steps from the complete genome to the GCF/MF pairs are indicated with the following scheme: Blue ovals = input and output data, green rectangles = actions with publicly available programs, purple rectangles = (currently) manual actions, diamond = decision, speech bubbles = additional information regarding the connected element. All manual actions followed strict logical rules that can be implemented for automatic analyses.

**
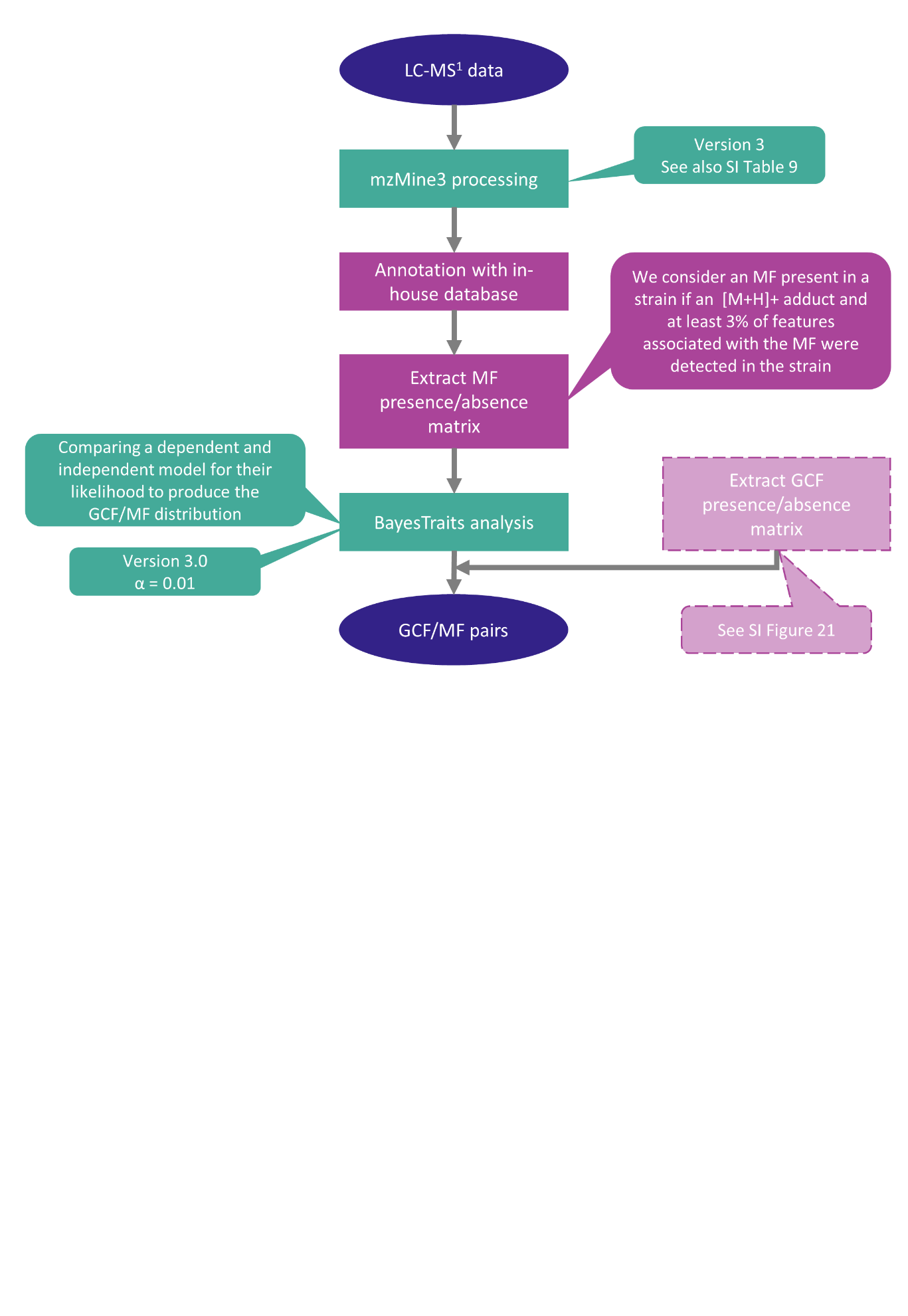
**

**SI Figure 22.** Metabolomic workflow of the metabologenomics approach. The individual steps from the LC-MS1 data to the GCF/MF pairs are indicated with the following scheme: Blue ovals = input and output data, green rectangles = actions with publicly available programs, purple rectangles = (currently) manual actions, speech bubbles = additional information regarding the connected element.
